# Does treatment with autophagy-enhancers and/or ROS-scavengers alleviate behavioral and neurochemical consequences of low-dose rotenone-induced mild mitochondrial dysfunction in mice?

**DOI:** 10.1038/s41380-023-01955-x

**Published:** 2023-01-23

**Authors:** O. Damri, S. Natour, S. Asslih, G. Agam

**Affiliations:** Psychiatry Research Unit and Department of Clinical Biochemistry and Pharmacology, Faculty of Health Sciences and Zlotowski Center for Neuroscience, Ben-Gurion University of the Negev, Beer-Sheva, Israel

**Keywords:** Bipolar disorder, Neuroscience

## Abstract

Bipolar-disorder’s pathophysiology and the mechanism by which medications exert their beneficial effect is yet unknown, but others’ and our data implicate patients’ brain mitochondrial-dysfunction and its amendment by mood-stabilizers. We recently designed a novel mouse bipolar-disorder-like model using chronic administration of a low-dose of the oxidative-phosphorylation complex I inhibitor, rotenone. Four and eight weeks rotenone treatment induced manic- and depressive-like behavior, respectively, accompanied by mood-related neurochemical changes. Here we aimed to investigate whether each of the autophagy-enhancers lithium (a mood-stabilizer), trehalose and resveratrol and/or each of the reactive oxygen species (ROS)-scavengers, resveratrol and N-acetylcystein and/or the combinations lithium+resveratrol or trehalose+N-acetylcystein, can ameliorate behavioral and neurochemical consequences of neuronal mild mitochondrial-dysfunction. We observed that lithium, trehalose and N-acetylcystein reversed rotenone-induced manic-like behavior as well as deviations in protein levels of mitochondrial complexes and the autophagy marker LC3-II. This raises the possibility that mild mitochondrial-dysfunction accompanied by impaired autophagy and a very mild increase in ROS levels are related to predisposition to manic-like behavior. On the other hand, although, as expected, most of the drugs tested eliminated the eight weeks rotenone-induced increase in protein levels of all hippocampal mitochondrial complexes, only lithium ubiquitously ameliorated the depressive-like behaviors. We cautiously deduce that aberrant autophagy and/or elevated ROS levels are not involved in predisposition to the depressive phase of bipolar-like behavior. Rather, that amending the depressive–like characteristics requires different mitochondria-related interventions. The latter might be antagonizing N-methyl-D-aspartate receptors (NMDARs), thus protecting from disruption of mitochondrial calcium homeostasis and its detrimental consequences. In conclusion, our findings suggest that by-and-large, among the autophagy-enhancers and ROS-scavengers tested, lithium is the most effective in counteracting rotenone-induced changes. Trehalose and N-acetylcystein may also be effective in attenuating manic-like behavior.

## Introduction

The pathophysiology of bipolar-disorder (BD) is not yet unraveled, but substantial evidence points at the involvement of mitochondrial-dysfunction [1–3]. In support of the latter, mood-stabilizers, the predominant treatment for BD, although discovered by serendipity and, as of now, with mechanism of action poorly understood, were shown to enhance mitochondrial-function and protect from oxidative-stress [4, 5], resulting in neuroprotection. Building on the involvement of mitochondrial-dysfunction in the etiology and treatment of BD we have recently designed a novel mouse model of BD-like behavior and neurochemistry [6] achieved by chronic treatment with a never used before low-dose rotenone, a mitochondrial complex-I (CoI) inhibitor, which induces mild mitochondrial-dysfunction.

Mitochondrial-dysfunction, sometimes caused by oxidative-stress and excess of reactive oxygen species (ROS), consequently leads to cell damage [7]. A rescue process which, most of the time, protects cells under these circumstances is autophagy/mitophagy [7–10]. Hence, here we reiterated our in vivo BD-like model and examined whether autophagy-enhancers and/or ROS-scavengers, comprised of either GRAS (generally regarded as safe) compounds or of approved mood-stabilizers, can fully, or at least partially, counteract rotenone-induced mitochondrial-dysfunction. We used drugs which we recently demonstrated to alleviate some rotenone-induced mild mitochondrial changes in SH-SY5Y cells [11].

The drugs included **I**. Autophagy-enhancers: (i) *lithium salts* (Li), the prototype mood-stabilizer used to treat BD patients [12–14]. In bipolar patients it induces anti-manic, anti-suicidal and prophylactic effects. In addition, it is used as adjunctive treatment to antidepressants in major depression [15, 16]. Several key cell signaling-related enzymes were found to be lithium-inhibitable, resulting in enhanced autophagy, elevated neurotrophic factors release, antioxidant effects, neuroinflammation and cytokine release attenuation and mitochondrial energy metabolism enhancement [17–20]. The involvement of these processes in a variety of neuropsychiatric disorders beyond BD raised, in the last decade, the interest in exploring Li’s potential neuroprotective property not only in psychiatric disorders but also in neurodegenerative and neurodevelopmental ones [17, 21–26].

(ii) *Trehalose*, a natural disaccharide comprised of two glucose molecules, is an autophagy modulator suggested as a treatment for neurodegenerative diseases in which autophagy has been shown to play a role [27–29]. In animal models trehalose was found to stimulate autophagy through the adenosine monophosphate–activated protein kinase (AMPK) [30], however, its role in autophagy is still controversial [31]. It functions as a stabilizer of proteins and is able to protect protein structural integrity. As previously reported by our lab, trehalose affected mouse brain autophagy markers in a manner indicative of enhanced autophagy, augmented manic-like behavior induced by amphetamine and attenuated immobility in the forced-swim test, the two latter effects being compatible with an anti-depressant-like effect [32]. Trehalose was also found to prevent neurodegeneration through decreasing damaged cell components [31].

**II**. A ROS-scavenger: *NAC* (N-Acetyl-L-cysteine) is a precursor of intracellular cysteine and of reduced glutathione (GSH) and is, thus, an inhibitor of ROS production, probably through conversion of NAC into hydrogen sulfide [33–35]. NAC improved neuronal deficits and nulled rotenone’s effect [36], increased midbrain dopaminergic neurons’ survival after rotenone treatment [37] and reduced methamphetamine-induced hyperthermia without affecting mice locomotor activity [38]. Clinical studies found a beneficial effect of NAC on the dopaminergic system in Parkinson’s disease patients, along with increased dopamine-transporter (DAT) binding in the caudate and the putamen [37, 39].

**III**. An autophagy-enhancer and a ROS-scavenger: *resveratrol* (RES, 3,5,4′-trihydroxy-trans-stilbene), a Sirt1 stimulator belonging to class-III histone deacetylases [40]. Resveratrol plays a role in autophagy regulation through mTOR inhibition [41, 42] and is also a ROS-scavenger [42–44]. As such, it inhibits excessive ROS production and lipid peroxidation, elevates mitochondrial membrane potential and inhibits cytochrome C release from the inner mitochondrial membrane, thus ameliorating aberrant mitochondrial distribution [45, 46]. Together, all resveratrol’s effects culminate, in the central nervous system (CNS), as follows. Reduced neurodegeneration in murine cerebral cortex and enhanced memory recovery following exposure to fluoride [46], as well as improved cognition, learning and memory in rats with vascular dementia and in mice models of Alzheimer’s disease [47].

Since we hypothesized that 1. Autophagy-enhancers and/or ROS-scavengers can fully, or at least partially, counteract/alleviate consequences of brain mild mitochondrial- dysfunction; 2. Combined treatment with an autophagy-enhancer and a ROS-scavenger is superior than each of them by its own in counteracting/alleviating consequences of brain mild mitochondrial-dysfunction, we also studied the effects of two pairs of drugs, lithium+resveratrol and trehalose+NAC on rotenone-induced mild mitochondrial-dysfunction as reflected in behavioral and neurochemical characteristics.

## Methods

### Animals

Male, five or eight weeks old mice (Envigo, Israel) were used. Animals were maintained on a 12 h/12 h light/dark cycle (lights on 7:00 a.m. to 7:00 p.m.) with constant temperature at 23 ± 1 ^o^C and *ad-libitum* access to food and water. Tests were performed during the light phase of the cycle between 9:00 am and 7:00 pm. Mice were allowed to acclimatize to the new environment for one week before treatment initiation. Animals were weighed once every third day and their general well-being assessed by fur examination and general appearance. No randomization method to allocate mice to a specific group/treatment was used. Blindness of the experimenter was kept during all experiments by the fact that the Ethovision software (see below) is independent of the experimenter. All experimental procedures followed the Israeli guidelines for treatment and care of experimental animals and were approved by the Ben-Gurion University animal experimentation ethics committee (Authorization Numbers: IL-50-07-2015 & IL-40-06-2019).

### Drugs’ delivery

Rotenone, dissolved in saline supplemented with 0.5% DMSO, was injected subcutaneously (s.c.), 0.75 mg/kg once daily for four or eight weeks. Control mice were injected with vehicle (saline supplemented with 0.5% DMSO). D-Amphetamine (Sigma-Aldrich, St Louis, MO) dissolved in saline, was injected intraperitoneally (i.p.), 1.5 mg/kg. NAC (Sigma-Aldrich, ibid) was administered in the drinking water, 1 g/kg body weight/day [48]. Trehalose (Sigma-Aldrich, ibid) was administered in drinking water, 0.2 g/kg body weight/day - 2% in drinking water. NAC and trehalose were administered either each separately or both together for two weeks either on their own or for the last two weeks to mice treated with rotenone for four or eight weeks. Resveratrol (Sigma-Aldrich, ibid), dissolved in 10% ethanol, was injected i.p. daily for 7 days, 15 mg/kg/day. Lithium (Sigma-Aldrich, ibid) was administered as LiCl in regular rodent chaw in powder form (Envigo, Israel), 0.2% w/w for five days followed by 0.4% for additional 10 days. Resveratrol and lithium were administered either each separately or both together, resveratrol - for the last week of rotenone treatment, and lithium - for the last two weeks of rotenone treatment.

### Behavioral experiments

#### Open field test

Mice were placed individually in the center of a transparent Plexiglas box (40 cm × 40 cm × 40 cm) for 20 min and their behavior digitally recorded. Amount and distribution of activity during the session was analyzed using an automated aquisition and analysis software (either Viewer, BiobServe, Bonn, Germany or the Noldus EthoVision system (Wageningen, the Netherlands).

#### Rotarod test

The rotarod test is based on a rotating rod which forces motor activity. It measures parameters such as riding time (seconds). The duration that mice stay on the rotating rod is a measure of their balance, coordination, physical condition, and motor-function. Animals were subjected to a series of one-minute sessions separated by ten minutes on the rotaroad apparatus as described: 0 rpm, 4 rpm, and three sessions of accelerating from 4 rpm to 40 rpm (two training sessions followed by the test session). Latency to fall was counted for each session.

#### Forced-swim test (FST)

The FST is a model of hopelessness (depressive)-like behavior. Mice were placed for a six min session in a transparent glass cylinder (22 cm diameter, 30 cm high) filled with water at 22 ± 1 °C so that the mouse cannot touch the bottom or climb out of the cylinder. Sessions are digitally recorded and the duration of immobility during the last four minutes of the six-min session automatically scored (FST, BiobServe, ibid or Noldus EthoVision system, ibid) for active (swimming or struggling) versus passive (floating, immobility). Immobility/floating is defined as the time spent by a mouse either motionless or making only those movements necessary to keep its head above water.

#### Elevated Plus-Maze Test (EPM)

The EPM models anxiety-like behavior; it is based on the animal’s aversion to open spaces, thus, spending more time in the enclosed arms. Mice were placed in a plus-shaped maze, elevated 50 cm above ground with four Plexiglas arms (5 × 30 cm each), two open ones with a 1 cm lip, and two closed ones with 24 cm height walls. Behavior was digitally recorded for a six min session and analyzed by a Viewer software (BiobServe/Noldus EthoVision system ibid) for the number of entries and time spent in the two kinds of arms. Data was used for computation of time in the open arms/total.

#### Sweet solution preference test (SSPT)

The test models hedonic-like behavior; namely, rodents are born with an interest in sweet foods and solutions. Reduced preference for sweet solution in the test represents anhedonia, increased consumption - hedonia. On top of the regular supply of water and food mice were supplied with a bottle of 1% saccharin solution (Sigma, ibid) for 48 h. Saccharin concentration was selected as it is in the high-end of the concentration-intake curve [49]. The saccharin solution bottle was made available to the mice throughout the entire test period. Weights of saccharin solution and water bottles were taken at the beginning of the experiment and every 24 h thereafter (a total of 3 measurements). Sweet solution preference was calculated daily as the ratio between amount of saccharin solution drank out of total liquid consumption.

#### Amphetamine-induced-hyperactivity/hyperlocomotion test (AIHT)

The test models manic-like behavior. Mice were placed individually in the center of a transparent Plexiglas box, as described for the open field test, and their behavior digitally recorded for 20 min (habituation period) following which they were injected with amphetamine (ip, 1.5 mg/kg) and placed back in the center of the same transparent Plexiglas box and further monitored by videotaping for an additional hour. Their behavior was analyzed as described above for the open-field test.

### Western blotting

Western blotting was performed according to a standard protocol used in our lab [50] on 10% acrylamid gel and transferred to PVDF membrane. Each sample was tested in duplicates of 10 and 20 µg total protein/lane, to verify linearity. Primary antibodies and their dilutions in TBST were: total OXPHOS cocktail (1:1500, Abcam, Cambridge Science Park, Cambridge, UK, cat. No. AB-ab 110413), p62 (1:1500, Abcam, ibid, cat. No. AB-ab 91526) and LC3-II (1:1000, Sigma-Aldrich, ibid, cat. No. L 7543). Secondary goat-anti rabbit antibodies (1:10000, Santa Cruz Biotechnology, Texas, USA, cat. No. sc2004) were also diluted in TBST. Results were normalized to Ponceau staining (Sigma-Aldrich, ibid) of total protein [51].

### Statistical analysis

Results are given either as means ± SEM of the original values or as means of % of control±SEM. Results exceeding + /−2SDs were excluded. Statistical analysis was carried out either by two-way ANOVA or by repeated measures ANOVA as appropriate and indicated for each analysis, followed by post-hoc Fisher’s Least Significant Difference (LSD) test, using STATISTICA software version 13 (StatSoft, Tulsa, Oklahoma). The data met the assumptions of the tests, namely, normal distribution and the variance between the compared groups was similar. *p* ≤ 0.05 was considered statistically significant. As a rule of thumb, according to our years of experience, and as can be found in others’ recent reports in a high impact journal [52, 53] a minimal number of animals or samples/group to achieve reproducibility is 5.

## Results

Based on our recently established low-dose rotenone–induced bipolar-like mouse phenotype with construct, face and predictive validity [6], the goal of the present study was to investigate whether in-vivo treatment with autophagy-enhancers and/or ROS-scavengers can fully, or at least partially, attenuate behavioral and neurochemical consequences accompanying the mild mitochondrial-dysfunction. The paradigm chosen was constructed to mimic the situation in real life. Namely, first, administration of rotenone for four or eight weeks, which induces the two disease-like states, and, thereafter, remedy is sought by drug administration. To this end, each of the autophagy-enhancers/ROS-scavengers (or combinations of them) were administered for the last either one week (resveratrol) or two weeks (lithium, trehalose and NAC) of rotenone treatment. As controls, the drugs were also administered for the same time periods by themselves (without rotenone). The results of the latter experiments are summarized under Supplementary table 1.

### Behavioral results

#### Open field and rotarod tests

We replicated our recent report [6] that 0.75 mg/kg/day rotenone by itself (administered for either four or eight weeks) does not affect the mice’ performance in the two paradigms (data not shown). Similarly, by-and-large, the autophagy-enhancers, the ROS-scavengers and their combinations had no influence neither on the distance travelled in the open field nor on the time to fall from the rotarod (data not shown).

#### FST

**Fig. 1:**
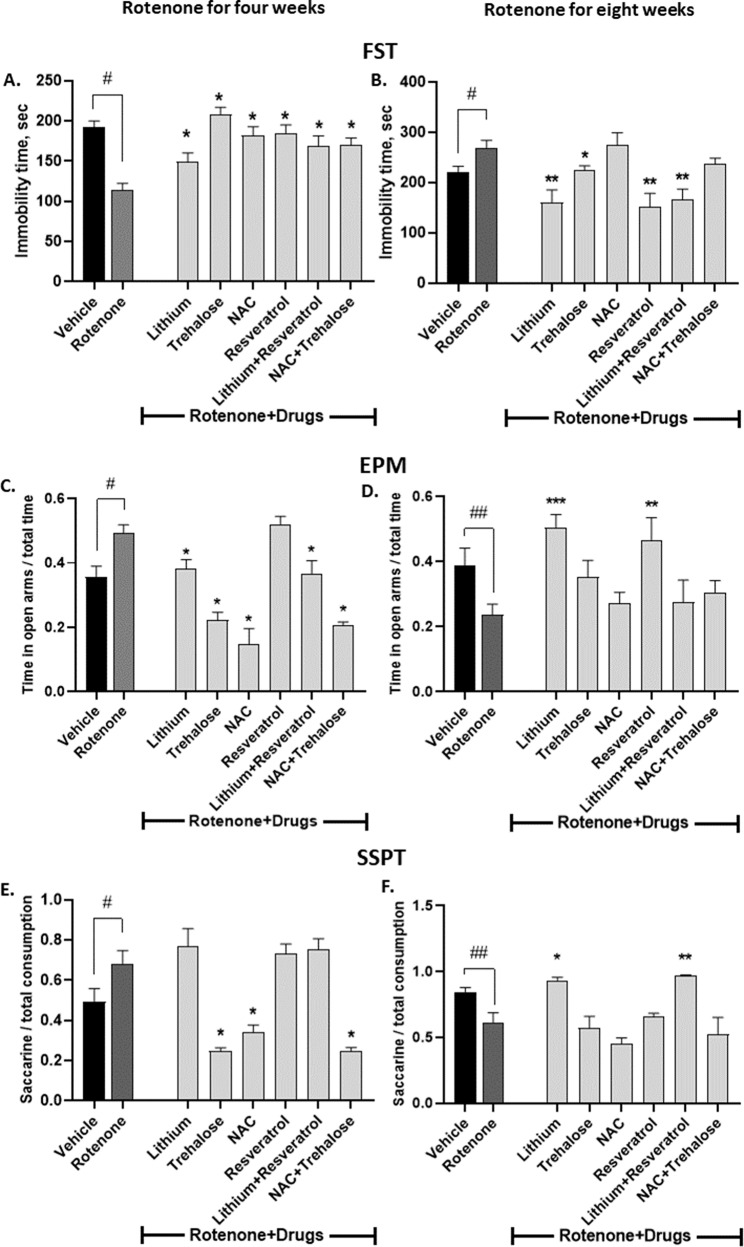
Some of the drugs and some of their combinations administered during the last week/two weeks of rotenone treatment for four (A, C, E) or eight (B, D, F) weeks attenuated rotenone’s effect in the FST, EPM and SSPT. Results represent means ± SEM of at least 7 mice/group. Our previous results [6] that four and eight weeks of rotenone treatment decreases and increases, respectively, the immobility time of the mice in the FST (**A**, **B**), and increases and decreases, respectively, the time spent in the open arms of the EPM (**C**, **D**), increases and decreases, respectively, the preference of the mice for sweet solution (**E**, **F**), and that lithium treatment attenuates rotenone’s effects in the FST and the EPM (**A**–**D**) were replicated. Two-ways ANOVA with rotenone (with/without) and drugs (with/without) as main factors: **FST - A**. rotenone effect, F(6,209) = 7.274, *p* < 0.0001; rotenoneXdrugs interaction, F(6,09) = 7.966, *p* < 0.0001. **B** Rotenone effect, F(6,111) = 13.21, *p* < 0.0001; **r**otenoneXdrugs interaction, F(6,111) = 2.557, *p* = 0.02**. EPM. C** Rotenone effect, F(6,166) = 27.13, *p* < 0.0001; **r**otenoneXdrugs interaction, F(6,166) = 6.234, *p* < 0.0001; drugs effect, F(1,166) = 8.449, *p* = 0.0042. **D** Rotenone effect, F(6,114) = 5.31, *p* = 0.0001; rotenoneXdrugs interaction, F(6,114) = 2.978, *p* < 0.01. **SSPT**. **E** Rotenone effect, F(6,81) = 16.53, *p* < 0.0001. **F** Rotenone effect, F(6,36) = 6.856, *p* < 0.0001; drugs effect, F(1,36) = 11.42, *p* = 0.0018. Asterisks and symbols denote results of Fisher’s LSD post-hoc test: ^#^*p* < 0.05 and ^##^*p* < 0.001 - *vs*. vehicle; *p < 0.05, **p < 0.001, and ***p < 0.0001 – *vs*. rotenone.

Fig. 1A, B summarizes the results. As we recently reported, 0.75 mg/kg/day rotenone for four or eight weeks significantly reduced or increased, respectively, the immobility time of the mice in the FST and lithium treatment for the last two weeks of rotenone attenuated both poles of bipolar-like behavior [6]. Rotenone-induced manic-like behavior (following four weeks of treatment) was fully counteracted by all the rest of the drugs and by their combinations (Fig. 1A). Rotenone’s eight weeks-induced effect on the performance of the mice in the FST was attenuated, in addition to lithium, by trehalose, resveratrol and lithium+resveratrol (Fig. 1B).

#### EPM

Fig. 1C, D summarizes the results. In a similar manner to the FST results, and as we recently reported, low-dose rotenone for four or eight weeks significantly increased or reduced (manic- and depressive-like behaviors), respectively, the duration proportion the mice spent in the open arms. Lithium treatment for the last two weeks of rotenone counteracted this bipolar-like behavior [6]. In addition to lithium, trehalose, NAC and both combinations (lithium+resveratrol and NAC + trehalose) also significantly abolished rotenone’s effect for four weeks (Fig. 1C). In the eight-weeks rotenone experiment (Fig. 1D), in addition to lithium, resveratrol also significantly counteracted rotenone’s effect and trehalose tended to do so without reaching statistical significance. The combinations were ineffective.

#### SSPT

Fig. 1E, F first depicts replication of our recent report that low-dose rotenone administration for four or eight weeks significantly increased or decreased, respectively, mice’ hedonic-like behavior (saccharine solution preference) [6]. Trehalose, NAC and their combination significantly obliterated rotenone’s four weeks effect (Fig. 1E) while lithium and lithium+resveratrol significantly counteracted rotenone’s eight weeks effect (Fig. 1F).

#### AIHT

**Fig. 2:**
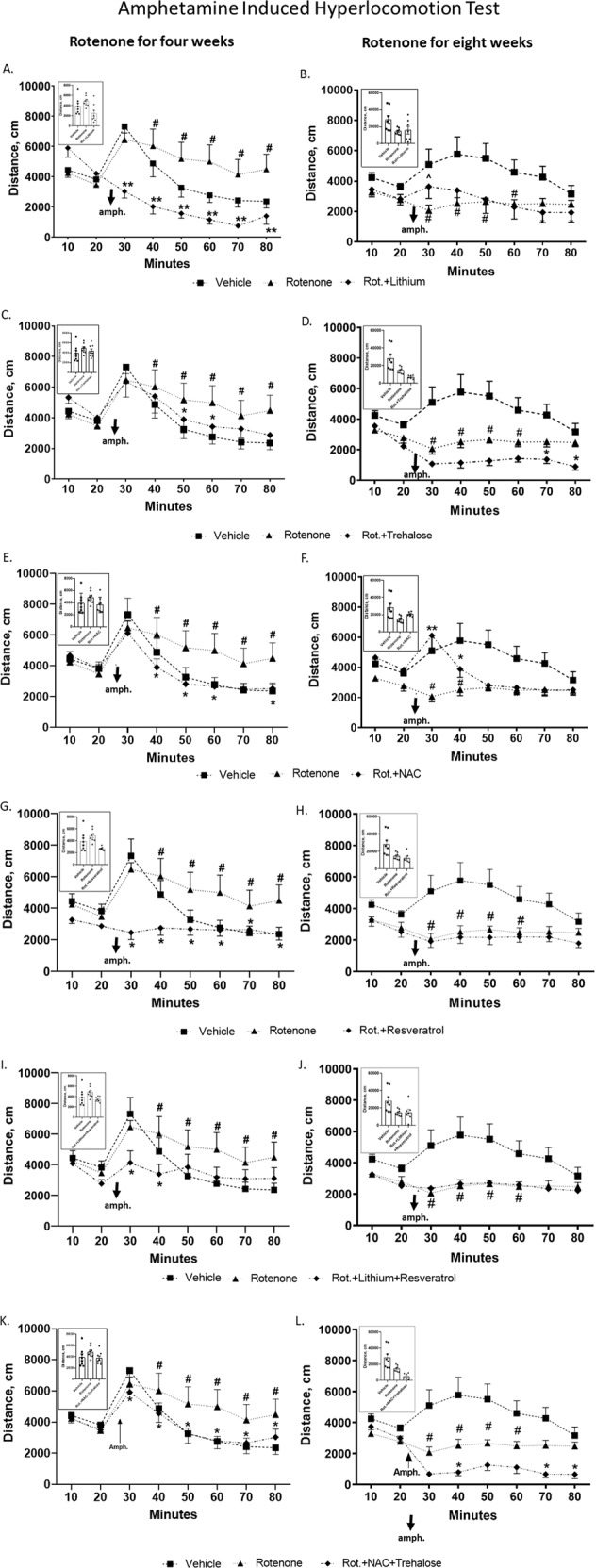
All drugs and their combinations administered during the last week/two weeks of rotenone treatment for four (A, C, E, G, I, K) or eight (B, D, F, H, J, L) weeks attenuated rotenone’s effect in the amphetamine-induced hyperlocomotion test, while lithium and NAC counteracted it in a time-dependent manner. Results represent means ± SEM of at least 7 mice/group. The plots depict distance travelled in the open field as a function of time; insets depict total distance travelled post amphetamine (amph.). As previously reported [6], repeated measures ANOVA of the results from 10 min post amphetamine injection to mice treated for four or eight weeks of rotenone, revealed significant augmentation and significant attenuation, respectively, of the distance travelled by the mice. Two-ways ANOVA with rotenone (with/without) and drugs (with/without) as main factors followed by Fisher’s LSD post-hoc test; asterisks and symbols denote: ^#^*p* < 0.05 and ^##^*p* < 0.001 - *vs*. vehicle; *p < 0.05, **p < 0.001 and ^*p* = 0.06 – *vs*. rotenone. Only statistically significant results are detailed: **Lithium** **A** Rotenone effect, F(7,189) = 21.236, *p* = 0.00001; rotenoneXdrugs interaction, F(21,189) = 6.304, *p* = 0.000001; drugs effect, F(3,27) = 4.191, *p* = 0.014; **B** Rotenone effect, F(7,182) = 7.784, *p* = 0.00001; rotenoneXdrugs interaction, F(21,182) = 2.196, *p* = 0.002; drugs effect, F(3,26) = 0.045, *p* = 0.017; **Trehalose** **C** Rotenone effect, F(7,168) = 22.382, *p* = 0.0001; rotenoneXdrugs interaction, F(21,168) = 1.774, *p* = 0.061. **D** Rotenone effect, F(7,168) = 5.487, *p* = 00001; rotenoneXdrugs interaction, F(21,168) = 2.437, *p* = 0.0009; drugs effect, F(3,24) = 0.194, *p* = 0.0003. **NAC** **E** Rotenone effect, F(7,168) = 26.308, *p* = 0.00001; rotenoneXdrugs interaction, F(21,168) = 1.916, *p* = 0.012. **F** Rotenone effect, F(7,168) = 2.960, *p* = 0.005; rotenoneXdrugs interaction, F(21,168) = 1.774, *p* = 0.024; drugs effect, F(3,24) = 1.063, *p* = 0.018. **Resveratrol** **G** Rotenone effect, F(7,189) = 7.186, *p* = 0.000001; rotenoneXdrugs interaction, F(21,189) = 5.332, *p* = 0.000001; drugs effect, F(3,27) = 4.433, p = 0.017; **H** Rotenone effect, F(7,182) = 3.299, *p* = 0.002; rotenoneXdrugs interaction, F(21,182) = 2.025, *p* = 0.007; drugs effect, F(3,26) = 0.84, *p* = 0.001; **Lithium** + **Resveratrol** **I** Rotenone effect, F(7,196) = 12.522, *p* = 0.000001; rotenoneXdrugs interaction, F(21,196) = 2.697, *p* = 0.0001; drugs effect, F(3,28) = 0.814, *p* = 0.057; **J** Rotenone effect, F(7,182) = 2.872, *p* = 0.007; rotenoneXdrugs interaction, F(21,182) = 1.789, *p* = 0.022; drugs effect, F(3,26) = 0.984, *p* = 0.007; **NAC** + **Trehalose** **K** Rotenone effect, F(7,161) = 23.703, *p* = 0.00001; **L**. rotenone effect, F(7,168) = 8.684, *p* = 0.000001; rotenoneXdrugs interaction, F(21,168) = 3.966, *p* = 0.000001; drugs effect, F(3,24) = 0.481, *p* = 0.00007.

Fig. 2A–L summarizes the results. As we recently reported, 0.75 mg/kg/day rotenone for four (Fig. 2A, C, E, G, I, K) or eight (Fig. 2B, D, F, H, J, L) weeks significantly augmented or reduced, respectively, amphetamine’s effect on the distance travelled by the mice in the open field, and lithium treatment for the last two weeks of rotenone’s four weeks administration significantly attenuated the hyperlocomotion induced by rotenone [6]. In addition to lithium all the drugs and the NAC + trehalose combination significantly obliterated rotenone’s four weeks treatment effect at most time points (Fig. 2A, C, E, G, I, K). Lithium and NAC attenuated rotenone’s eight weeks administration effect on amphetamine-induced-hyperlocomotion at sporadic time points (Fig. 2B, F). Unexpectedly, NAC + trehalose further downregulated amphetamine’s effect beyond that of rotenone (Fig. 2K, L).

### Neurochemical results - hippocampal protein levels of the mitochondrial respiration complexes

#### Complexes (Co)s I-V

The pattern of reduced and increased hippocampal protein levels of the complexes following four and eight weeks of rotenone, respectively, was replicated [6].

#### CoI

**Fig. 3:**
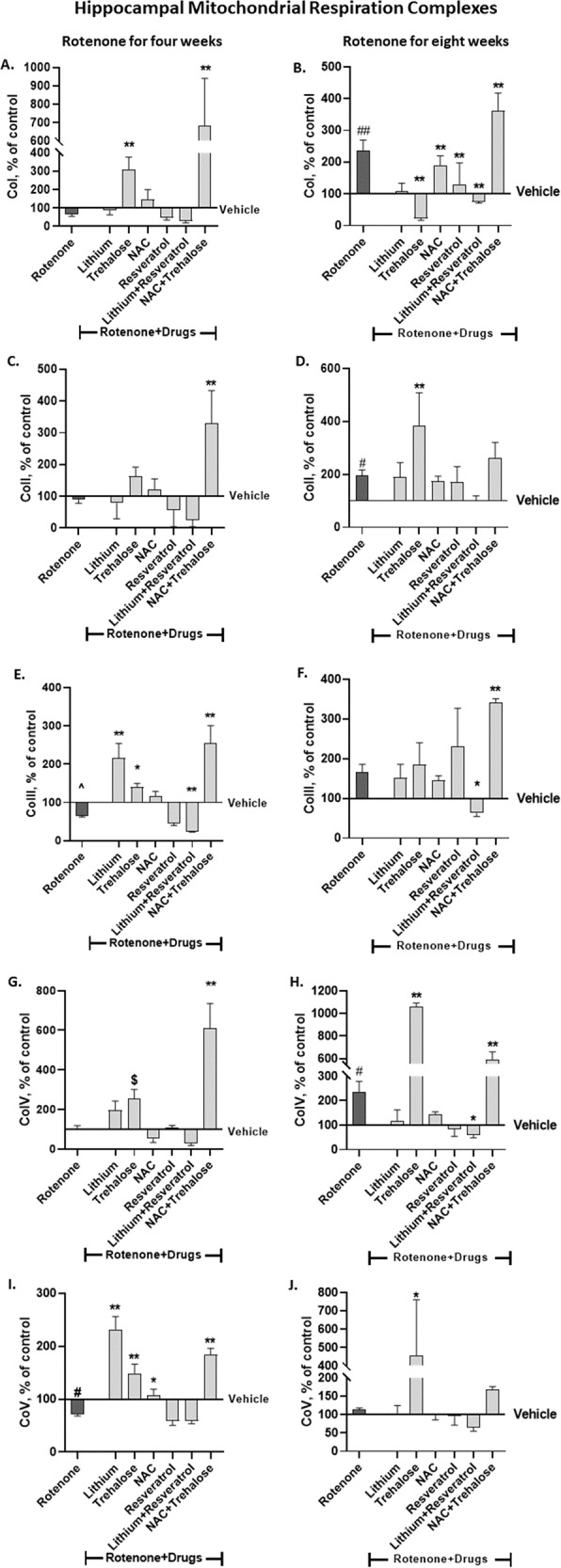
The effect of rotenone for four (A, C, E, G, I) and eight weeks (B, D, F, H, J) on hippocampal protein levels of the five mitochondrial respiration complexes was attenuated or counteracted by some of the drugs or their combination administered during the last week/two weeks of rotenone treatment. Results, expressed as percent of vehicle only, represent means ± SEM of at least 6 mice/group. Our previous results [6] of the effect of rotenone treatment for four and eight weeks on hippocampal protein levels of the complexes were replicated. Two-ways ANOVA with rotenone (with/without) and drugs (with/without) as main factors followed by Fisher’s LSD post-hoc test; asterisks and symbols represent: ^#^*p* < 0.05, ^##^*p* < 0.001 and ^one-tailed *p* < 0.05 *vs*. vehicle; *p < 0.05, **p < 0.001 and ^$^one-tailed *p* < 0.05, *vs*. rotenone. Only significant results are detailed: **Co I – A** (4 weeks of Rot): rotenone effect, F(6,85) = 18.47, *p* < 0.0001. **B** (8 weeks of Rot): RotenoneXdrugs interaction, F(6,63) = 4.818, *p* = 0.0004; rotenone effect, F(6,63) = 19.54, *p* < 0.0001 **Co II – C** (4 weeks of Rot): rotenone effect, F(6,92) = 23.39, p < 0.0001**. D** (8 weeks of Rot): rotenone effect, F(6,69) = 8.014, p < 0.0001. **Co III – E**. (4 weeks of Rot): RotenoneXdrugs interaction, F(6,84) = 6.711, *p* < 0.0001; rotenone effect, F(6,84) = 34.67, *p* < 0.0001. **F** (8 weeks of Rot): RotenoneXdrugs interaction, F(6,68) = 3.167, *p* = 0.085; rotenone effect, F(6,68) = 16.89, *p* < 0.0001. **Co IV – G** (4 weeks of Rot): Rotenone effect, F(6,88) = 14.91, *p* < 0.0001. **H** (8 weeks of Rot): Rotenone effect, F(6, 62) = 56.13, *p* < 0.0001; **Co V - I** (4 weeks of Rot): RotenoneXdrugs interaction, F(6,89) = 8.891, *p* < 0.0001; rotenone effect, F(6,89) = 47.02, *p* < 0.0001. **J** (8 weeks of Rot): Rotenone effect, F(6, 68) = 6.45, *p* < 0.0001.

Trehalose and NAC + trehalose drastically and significantly counteracted and upregulated CoI protein levels following four weeks of rotenone (Fig. 3A). The effect of eight weeks rotenone was abolished by lithium, trehalose, resveratrol and the lithium+resveratrol combination (Fig. 3B).

#### CoII

Trehalose and the combination NAC + trehalose significantly revived hippocampal CoII protein levels following four weeks of rotenone treatment (Fig. 3C). Trehalose significantly augmented rotenone’s eight weeks effect (Fig. 3D).

#### CoIII

The trend of the reducing effect of four weeks rotenone was counteracted by lithium, trehalose and NAC + trehalose (Fig. 3E). In the eight weeks experiment only lithium+resveratrol counteracted the trend of the increasing effect of rotenone while NAC + trehalose significantly augmented rotenone’s effect (Fig. 3F). It may be noted that while rotenone by itself did not affect CoIII protein levels, rotenone with the combination lithium+resveratrol further decreased them, raising the possibility that this combination was toxic for CoIII.

#### CoIV

Four weeks of rotenone treatment did not affect hippocampal CoIV protein levels (Fig. 3G), trehalose and NAC + trehalose administration for the last two weeks of rotenone significantly increased them (Fig. 3G). The significant upregulation of hippocampal CoIV protein levels following eight weeks of rotenone administration was significantly counteracted by the lithium+resveratrol combination and non-significantly counteracted by lithium, NAC and resveratrol. Unexpectedly, trehalose and NAC + trehalose administration for the last two weeks of rotenone significantly upregulated CoIV levels beyond rotenone’s effect (Fig. 3H).

#### CoV

The significantly decreased hippocampal CoV protein levels induced by four weeks of rotenone administration was significantly counteracted by trehalose, NAC and NAC + trehalose (Fig. 3I). Rotenone administration for eight weeks had no effect on hippocampal CoV protein levels (Fig. 3J). In the eight weeks rotenone experiment trehalose administration for the last two weeks of rotenone significantly increased the levels compared to control and to rotenone (Fig. 3J).

### Neurochemical results – frontal-cortex protein levels of the mitochondrial respiration complexes

#### Cos I-V

As described above for the results in the hippocampus, by-and-large, in the frontal-cortex, the overall pattern of lack of effect of four weeks rotenone on the complexes’ levels (except for elevated CoIV levels) and increased levels following eight weeks of rotenone as we recently reported [6] was replicated.

#### CoI

**Fig. 4:**
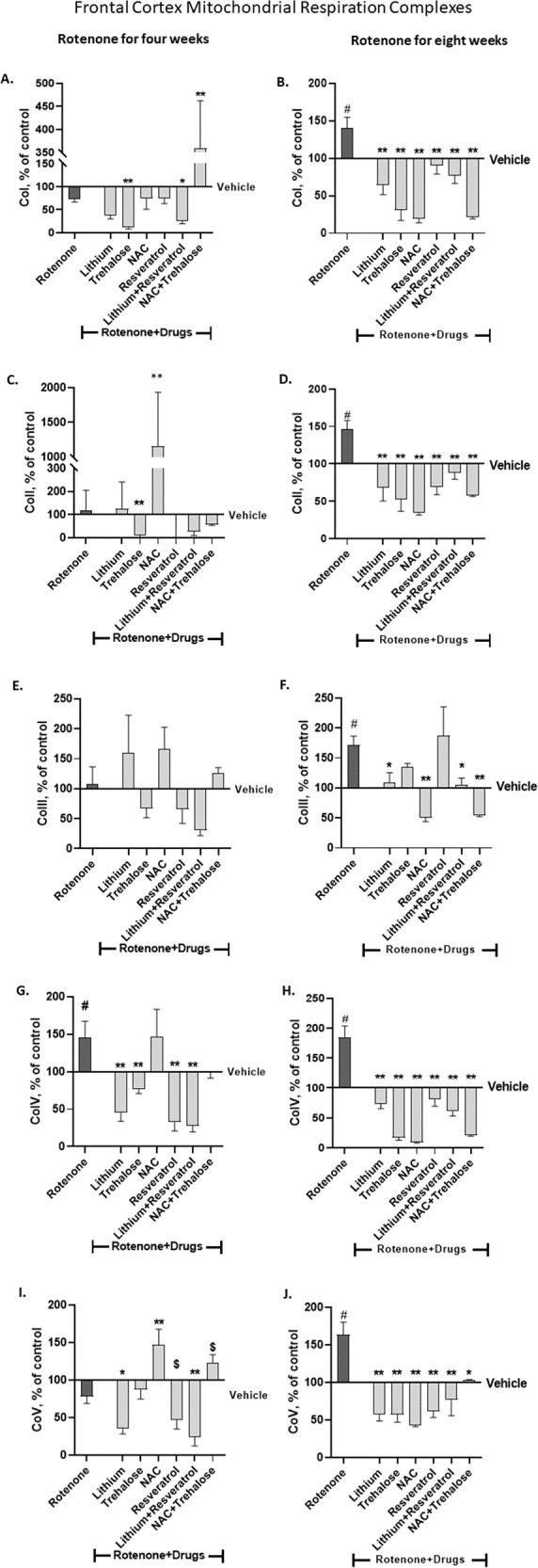
The effect of rotenone for four (A, C, E, G, I) and eight weeks (B, D, F, H, J) on frontal cortex protein levels of the five mitochondrial respiration complexes was attenuated or counteracted by some of the drugs or their combination administered during the last week/two weeks of rotenone treatment. Results, expressed as percent of vehicle only, represent means ± SEM of at least 6 mice/group. Our previous results of the effect of rotenone treatment for four and eight weeks on frontal cortex protein levels of the complexes [6] were replicated. Two-ways ANOVA with rotenone (with/without) and drugs (with/without) as main factors followed by Fisher’s LSD post-hoc test; asterisks and symbol represent: ^#^*p* < 0.05 vs. vehicle; *p < 0.05, **p < 0.001 and $one-tailed *p* < 0.05 vs. rotenone. Only significant results are detailed: **Co I – A** (4 weeks of Rot) rotenoneXdrugs interaction, F(6,67) = 16.64, *p* < 0.0001; rotenone effect, F(6,67) = 18.77, *p* < 0.0001. **B** (8 weeks of Rot) rotenoneXdrugs interaction, F(6,70) = 2.102, *p* = 0.06; rotenone effect, F(6,70) = 16.94, *p* < 0.0001. **Co II – C** (4 weeks of Rot) rrotenone effect, F(6,86) = 31.17, *p* < 0.000. **D** 8 weeks of Rot: rotenone effect, F(6,74) = 14.35, *p* < 0.0001. **Co III – E** (4 weeks of Rot) rrotenone effect, F(6,84) = 2.433, *p* = 0.0. **F** (8 weeks of Rot) rotenoneXdrugs interaction, F(6,72) = 4.632, *p* = 0.0005; rotenone effect, F(6,72) = 3.028, *p* = 0.0107. **Co IV – G** (4 weeks of Rot) rotenone effect, F(6,76) = 12.96, *p* < 0.000. **H** (8 weeks of Rot) rotenoneXdrugs interaction, F(6,70) = 6.341, *p* < 0.0001; rotenone effect, F(6, 70) = 32.8, *p* < 0.0001. **Co V - I** (4 weeks of Rot) rotenone effect, F(6,79) = 15.55, *p* < 0.0001. **J** (8 weeks of Rot) rotenoneXdrugs interaction, F(6,73) = 2.802, *p* = 0.016; rotenone effect, F(6, 73) = 10.81, *p* < 0.0001.

Rotenone administration for four weeks had no effect on frontal-cortex CoI protein levels (Fig. 4A). Trehalose administered for the last two weeks and lithium+resveratrol (resveratrol - for the last week, lithium - for the last two weeks) significantly decreased CoI protein levels while NAC + trehalose significantly increased them (Fig. 4A). Rotenone administration for eight weeks significantly increased frontal cortex CoI protein levels; all the drugs and their combinations significantly counteracted rotenone’s effect (Fig. 4B).

#### CoII

Four weeks of rotenone did not affect frontal-cortex CoII protein levels (Fig. 4C). Trehalose and NAC administered for the last two weeks of rotenone treatment significantly decreased and increased the levels, respectively (Fig. 4C). Rotenone administration for eight weeks significantly increased frontal-cortex CoII protein levels and all the drugs and their combinations counteracted this effect (Fig. 4D).

#### CoIII

Frontal-cortex CoIII protein levels were not affected, neither by four weeks of rotenone treatment nor by the drugs or their combinations (Fig. 4E). Rotenone administration for eight weeks significantly increased frontal-cortex CoIII protein levels (Fig. 4F). Lithium, NAC and both combinations significantly counteracted rotenone’s effect (Fig. 4F).

#### CoIV

Rotenone administration for either four or eight weeks significantly increased frontal-cortex CoIV protein levels (Fig. 4G, H). All the drugs and their combinations administered for the last one/two weeks of rotenone treatment significantly counteracted rotenone’s effect except for NAC which was not effective following rotenone treatment for four weeks (Fig. 4G, H).

#### CoV

Rotenone administration for four weeks did not affect frontal-cortex CoV protein levels (Fig. 4I). Lithium and lithium+resveratrol administered for the last week/two weeks of rotenone treatment significantly decreased CoV levels and NAC increased them (Fig. 4I). Rotenone administration for eight weeks significantly increased frontal-cortex CoV protein levels and all the drugs and their combination administered for the last week/two weeks of rotenone significantly counteracted rotenone’s effect (Fig. 4J).

### The effect of the autophagy-enhancers and the ROS-scavengers on rotenone’s effect on hippocampal and frontal-cortex levels of the autophagy-related proteins LC3-II and p62

#### Hippocampus - four weeks of rotenone treatment

**Fig. 5:**
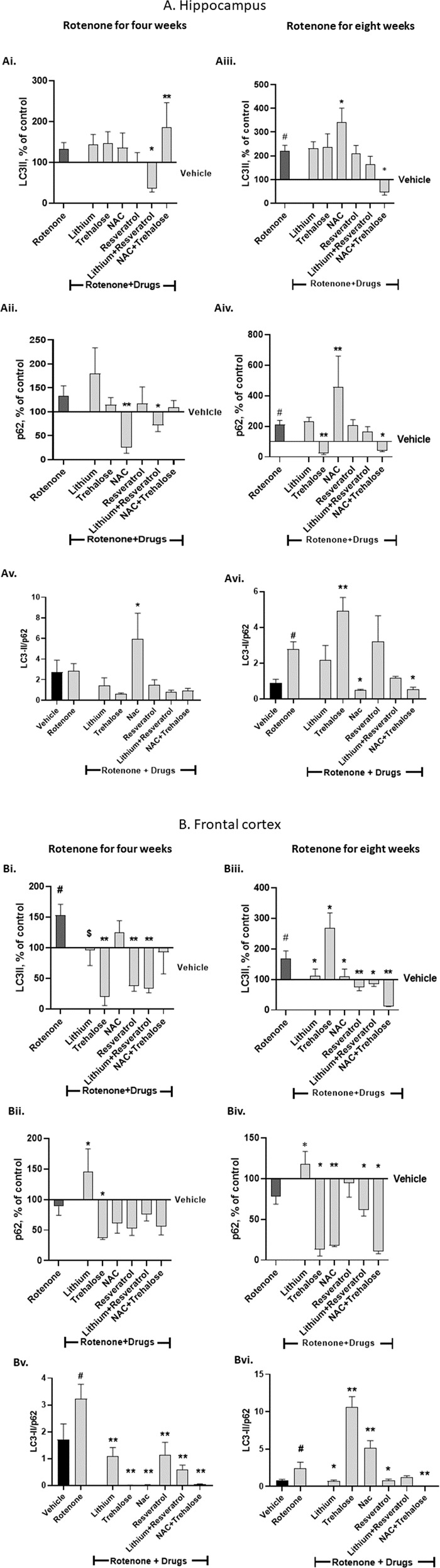
Hippocampal (A) and frontal cortex (B) protein levels of the autophagy markers LC3-II (Ai, Aiii, Bi, Biii), p62 (Aii, Aiv, Bii, Biv) and LC3-II/p62 protein levels ratio (Av, Avi, Bv, Bvi) - effect of the drugs tested on their own and of their combinations on rotenone’s effect for four (Ai, Aii, Av, Bi, Bii, Bv) and eight (Aiii, Aiv, Avi, Biii, Biv, Bvi) weeks. Results of LC3-II and p62, expressed as percent of vehicle only, and of the ratio between the arbitrary units of LC3-II and p62, represent means ± SEM of at least 5 mice/group. Two-ways ANOVA with rotenone (with/without) and drugs (with/without) as main factors followed by Fisher’s LSD post-hoc test; asterisks and symbol represent: ^#^*p* < 0.05 *vs*. vehicle; *p < 0.05, **p < 0.001 and ^$^one-tailed *p* < 0.05 *vs*. rotenone. Only significant results are detailed: **A Hippocampus** 4 weeks of rotenone: **Ai**. LC3-II: rotenone effect, F(6,69) = 6.59, *p* < 0.0001. **Aii** p62: rotenoneXdrugs interaction, F(6,67) = 16.64, *p* < 0.0001; rotenone effect, F(6,67) = 18.77, *p* < 0.0001. 8 weeks of rotenone: **Aiii** LC3-II: rotenone effect, F(6,65) = 10.99, *p* < 0.0001. **Aiv** p62: rotenone effect, F(6,72) = 11.55, *p* < 0.0001; drugs effect, F(1,72) = 0.717, *p* = 0.002. **Av** LC3-II/p62: drugs effect, F(6,64) = 5.04, *p* = 0.0003. **Avi** LC3-II/p62: rotenoneXdrugs interaction, F(6,55) = 5.007, *p* = 0.0004; drugs effect, F(6,55) = 4.541, *p* = 0.0008; rotenone effect, F(6,55) = 23.25, *p* = 0.0001. **B Frontal cortex** 4 weeks of rotenone: **Bi** LC3-II: rotenoneXdrugs interaction, F(6,75) = 3.794, *p* = 0.002; rotenone effect, F(6,75) = 2.618, *p* = 0.02. **Bii** p62: rotenone effect, F(6,83) = 4.267, *p* = 0.0009. 8 weeks of rotenone: **Biii** LC3-II: rotenoneXdrugs interaction, F(6,65) = 5.216, *p* = 0.0002; rotenone effect, F(6,65) = 12, *p* < 0.0001. **Biv** p62: rotenone effect, F(6,70) = 21.64, *p* < 0.0001. **Bv** LC3-II/p62: rotenoneXdrugs interaction, F(6,64) = 2.22, *p* = 0.05; drugs effect, F(6,64) = 9.521, *p* < 0.0001. **Bvi** LC3-II/p62: rotenoneXdrugs interaction, F(6,54) = 12.51, *p* < 0.0001; drugs effect, F(6,54) = 43.97, *p* < 0.0001; rotenone effect, F(6,54) = 32.04, *p* < 0.0001.

Rotenone did not affect hippocampal LC3-II and p62 protein levels (Fig. 5Ai, Aii). Among the drugs administered during the last weeks of rotenone only NAC + trehalose significantly elevated LC3-II levels (Fig. 5Ai) and only NAC and lithium+resvertatrol significantly decreased p62 levels (Fig. 5Aii). These results culminated in a significant elevation of the LC3-II/p62 ratio by NAC (Fig. 5Av).

#### Hippocampus - eight weeks of rotenone treatment

Rotenone significantly increased hippocampal LC3-II and p62 protein levels (Fig. 5Aiii, Aiv). NAC added during the last two weeks of rotenone augmented rotenone’s effect on both LC3-II and p62 (Fig. 5Aiii, Aiv) while trehalose and NAC + trehalose counteracted rotenone’s effect on p62 (Fig. 5Aiv). These results culminated in a significant elevation of the LC3-II/p62 ratio by trehalose and a significant counteraction of rotenone-induced elevation of the ratio by NAC and NAC + trehalose (Fig. 5Avi).

#### Frontal-cortex - four weeks of rotenone treatment

Rotenone treatment significantly elevated LC3-II protein levels (Fig. 5Bi). Lithium, trehalose, resveratrol and lithium+resveratrol significantly counteracted rotenone’s effect (Fig. 5Bi). Rotenone treatment did not affect p62 protein levels (Fig. 5Bii), yet, lithium and trehalose significantly elevated and downregulated, respectively, p62 levels (Fig. 5Bii). These results culminated in a significant elevation of the LC3-II/p62 ratio by rotenone, which was counteracted by all the drugs and their combinations (Fig. 5Bv).

#### Frontal-cortex - eight weeks of rotenone treatment

Rotenone treatment significantly elevated LC3-II protein levels (Fig. 5Biii). All drugs and combinations tested, except for trehalose, when administered for the last weeks of rotenone, significantly counteracted rotenone’s effect while trehalose augmented it (Fig. 5Biii). p62 protein levels were not affected by rotenone, but the non-significant trend of downregulation was counteracted by lithium (Fig. 5Biv). Trehalose, NAC, their combination and lithium+resveratrol administered during the last weeks of rotenone significantly decreased p62 protein levels (Fig. 5Biv). These results culminated in a significant elevation of the LC3-II/p62 ratio by rotenone, which was counteracted by lithium, resveratrol and NAC + trehalose, but further augmented by trehalose (Fig. 5Bvi).

*Original blot images are given in the Supplementary Material*

## Discussion

We based the present study on our recently established low-dose rotenone-induced manic- and depressive-like behavior when administered for four and eight weeks [6]. It corroborates the concept that BD involves gradual decrease in mitochondrial-function, with symptoms beginning only when a threshold is reached or when an event occurs which requires fully functional cells, making the subject more vulnerable to environmental factors targeting mitochondrial-function [54]. Under physiological conditions, dysfunctional mitochondria are degraded by the quality control pathway designated mitophagy - autophagy of mitochondria [55]. It is requiered for mitochondrial turnover and cellular homeostasis; under stress stimuli it is requiered to remove damaged mitochondria, preserving healthy mitochondrial functioning and, consequently, preventing further damage and cell death [56, 57]. One of the early responses to excessive ROS production is the induction of mitophagy. It has been suggested that aberrant cell function caused by oxidative-stress and mitochondrial-dysfunction may be ameliorated by enhancing mitophagy [24, 57–61]. ROS-scavengers also directly correct mitochondrial-dysfunction by decreasing intracellular Ca^+2^ levels [62, 63] and inhibiting apoptotic pathways [64]. Here we aimed to confirm or reject our hypothesis that autophagy-enhancers [lithium [65, 66] and trehalose [67]] and/or ROS-scavengers [NAC and resveratrol [45, 68]] can alleviate the in-vivo rotenone-induced behavioral and biochemical consequences of mild mitochondrial-dysfunction in mice.

Since, unfortunately, up until now, there is no prognostic tool to envisage one’s risk to become afflicted with BD, the design of the paradigm employed was meant to mimic this realm. Namely, induce the disease-like state and then try to relieve it.

As we previously reported [6], and unlike rotenone-induced Parkinson’s disease-like model [69, 70], our rotenone dose administered for four or eight weeks did not affect spontaneous and motor activity of the mice as reflected in the open field and the rotarod tests, and manic- and depressive-like behaviors were observed in the FST, EPM, SSPT and the AIHT. We now hypothesized that both autophagy-enhancers and ROS-scavengers may be beneficial for the treatment of BD and that combining an autophagy-enhancer and a ROS-scavenger might be superior than each of them by itself. Given that in our hands and our rotenone model ROS level change was not detected [6], one may wonder why we chose ROS scavengers for treatment of rotenone-induced mild mitochondrial changes. Our rationale was as follows. Rotenone treatment induces mitochondrial homeostasis imbalance which might, initially, result in upregulated ROS levels. However, this, in turn, induces autophagy upregulation which, among a myriad of consequences, scavenges ROS [71]. Since we are monitoring chronic rotenone effects (following four and eight weeks of treatment), we, possibly, ‘miss’ ROS levels upregulation. Yet, treatment with ROS scavengers and/or autophagy enhancers intervenes with mitochondrial homeostasis which has been interrupted by rotenone and, possibly, counteracts or attenuates the interruption. Our hypothesis was partially confirmed. Namely, each of rotenone-induced mood-related behaviors was counteracted/attenuated by some of the drugs and some of the combinations, but the effect of the combinations was not superior than the drugs on their own. Interestingly, lithium was superior as compared with the rest of the tested drugs. As we already recently reported [6], lithium counteracted rotenone’s effects in the majority of the mood-related behavioral tests as could be expected based on lithium’s well-established amelioration of anxiety- and depressive-like behavior [72, 73], and the drug’s anti-manic, anti-depressive and anxiolytic effect in the clinics [74]. In particular, the depressive-like behavior induced by eight weeks of rotenone was ubiquitously attenuated only by lithium whereas trehalose and resveratrol were effective only in the FST and the EPM, lithium+resveratrol – in the FST and the SSPT and NAC + trehalose – in the AIHT. This is compatible with our previous results in human neuronal cells (SH-SY5Y) exposed to a low rotenone concentration for 72 and 96 h, which exhibited up- and down-regulation of mitochondrial respiration, respectively [11]. In the neuronal cells we studied whether the autophagy-enhancers lithium, trehalose, rapamycin, and resveratrol and/or the ROS-scavengers [resveratrol, NAC and Mn-Tbap [Mn(III)tetrakis (4-benzoic acid) porphyrin)] can ameliorate neuronal mild mitochondrial-dysfunction. Only lithium (added for the last 24/48 h of the exposure to rotenone for 72/96 h, respectively) counteracted the effect of rotenone on most of the mitochondrial respiration parameters [measured as oxygen consumption rate (OCR)] in both paradigms. Rapamycin, resveratrol, NAC and Mn-Tbap counteracted most of rotenone’s effects on OCR-parameters only after 72 h [11], possibly *via* different mechanisms, not necessarily related to their autophagy enhancement and/or ROS scavenging effects. Lithium-induced reversal of rotenone on OCR-parameters is compatible with the drug’s known positive effects on mitochondrial-function [75–77], possibly mediated *via* its effect on autophagy/mitophagy [75, 78]. Indeed, Osete et al. [76] recently reported amelioration of rotenone-induced mitochondrial respiration malfunction (measured as OCR) in iPSCs (induced pluripotent stem cells) derived neural precursor cells by lithium. The lack of consistency in the results with the other autophagy-enhancers and/or ROS-scavengers may, cautiously, suggest that lithium’s antidepressant effect is not mediated *via* these mechanisms.

It is well established that up- and down-regulation of LC3-II and p62, respectively, may be interpretable as enhanced autophagy [79, 80]. In parallel with our finding in the cell model of rotenone-induced mild mitochondrial-dysfunction [11], the lack of elevation and even significantly decrease in LC3-II following the administration of the mTOR inhibitor resveratrol for the last week of the exposure to rotenone for either four or eight weeks might be explained by the report that under mitochondrial complex I defects mTOR inhibitors suppress autophagy [81]. Trehalose, on the other hand, enhances autophagy not by mTOR inhibition and, indeed, compatible with our report in neuroblastoma cells [11], here it elevated autophagy following eight weeks of rotenone treatment.

As previously reported by our lab trehalose by itself augmented manic-like behavior induced by amphetamine [32]. When administered for the last two weeks of four-weeks rotenone treatment, trehalose abolished rotenone’s manic-like effects, while in the depressive-like behavior it did not counteract rotenone effects except for reducing immobility time in the FST as also reported by others in a genetic model of diabetes with signs of neuronal damage [82].

Manic-like behavior induced by four weeks of rotenone was counteracted by two weeks of NAC treatment as reflected in the FST, the EPM and the AIHT. The results provide credentials to the suggestions of NAC as a candidate for the treatment of BD and schizophrenia [83, 84]. As expected, lithium+resveratrol and NAC + trehalose significantly counteracted rotenone-induced most manic-like effects. This suggests that at least in the case of mania combining forces of autophagy enhancement and ROS scavenging might help in lowering the drugs’ dose and, thus, reduce side effects. This is in contrast with the depressive-like behaviour induced by eight weeks of rotenone treatment which was not reversed by each of trehalose or NAC or by their combination.

The present resveratrol results are similar to the report that the drug decreased anxiety-like and depressive-like behaviors in diabetic rats [85]. Sahin and colleagues suggested that this action stemmed from normalizing nitric-oxide (NO) levels. However, this is not compatible with the current results since, in our hands, NAC (also a ROS-scavenger) did not exhibit the same results in the FST and the EPM experiments.

To conclude the behavioral results – (i) We replicated our recent report [6] that mildly impaired mitochondrial-function following low-dose rotenone treatment induces behavioral changes representing various facets of BD. (ii) The observation that lithium, trehalose (an autophagy-enhancer) and NAC (a ROS-scavenger) reversed rotenone-induced manic-like behavior raises the possibility that mild mitochondrial-dysfunction accompanied by impaired autophagy and a very mild increase in ROS levels are related to predisposition to manic-like behavior. (iii) Despite pointing at a causality relationship between mild mitochondrial-dysfunction and depressive-like symptoms, only lithium, but not the other drugs, ubiquitously ameliorated the rotenone-induced depressive-like behaviors. We, therefore, cautiously deduce that aberrant autophagy and/or elevated ROS levels are not involved in the predisposition to the depressive phase of bipolar-like behavior. Rather, abnormally activated N-methyl-D-aspartate receptors (NMDARs), essential in synaptic plasticity and excitotoxicity, may be relevant. The rationale being as follows: lithium has been repeatedly implicated as an inhibitor of these receptors [86, 87]; As recently reviewed by Corriger and Pickering [88] and by Jelen and stone [89] ketamine, an NMDAR antagonist, exhibits a rapid antidepressant property after a single or repetitive sub-anesthetic dose in individuals with treatment-resistant major depressive disorder [90] and BD [91]. As for lithium- and ketamine-induced side effects, the NMDAR/NO system has been suggested to be involved in some adverse effects of lithium, including diabetes insipidus, erectile dysfunction, vascular function, and gastrointestinal dys-motilities [92]. However, to the best of our knowledge, the lithium-induced side effects hypothyroidism and renal insufficiency which appear following years of treatment [93], have not been attributed to the drug’s effect on NMDARs. Ketamine-induced side effects, including psychiatric (other than the primary antidepressant outcome), psychomimetic, neurologic and cardiovasculr ones [94] are regarded to stem from the drug’s effect on NMDARs [95]. Intriguingly, in relation with the involvement of mitochondrial dysfunction in BD, in general, and in bipolar depression, in particular, a link has recently been suggested between NMDAR-mediated excitotoxicity and mitochondrial dysfunction [96]. The authors build their case on the fact that the NMDAR is a calcium channel, that calcium entry through extrasynaptic NMDARs is linked to calcium overload in the mitochondria in neurons, and that disruption of mitochondrial calcium homeostasis is linked to neuronal death by triggering apoptosis or by opening of the mitochondrial transition pore.

At the molecular level, for obvious relevance, we addressed protein levels of the five mitochondrial respiration complexes and two autophagy markers in the hippocampus and frontal-cortex, two brain areas robustly implicated in BD [97–99]. As mentioned above, we hypothesized that the mild mitochondrial-dysfunction induced by our selected low rotenone regime can fully, or at least partially, be counteracted by autophagy-enhancers and/or ROS-scavengers. Compatible with the different functionality of these two brain regions [100, 101], a variety of biochemical results, both those of rotenone’s effects and the capability of the autophagy-enhancers and ROS-scavengers to counteract them, also differed between them.

It is noteworthy that the pattern of rotenone’s effect on the hippocampal and frontal-cortex protein levels of the five mitochondrial respiration complexes was very similar to that of our recent study [6]. Furthermore, similarly to a recent report [102] that exposure of *C. elegans* for 24 and 48 h to 1 or 5 μM rotenone upregulated mitochondrial complexes II and V without major changes in oxygen-consumption or steady-state ATP levels, we report rotenone-induced increased frontal-cortex CoII and CoV and hippocampal CoII levels following eight weeks of treatment.

Among the five mitochondrial respiration complexes, rotenone treatment for four weeks exerted a statistically significant effect only on hippocampal CoV and frontal-cortex CoIV protein levels, reducing the former and elevating the latter. The same rotenone regime also upregulated frontal-cortex LC3-II levels and LC3-II/p62 protein levels ration, indicative of autophagy upregulation as previously reported by us [32, 103] and recently advised by Klionski et al. [80] for the Beclin 1/p62 ratio or its reciprocal, and previously reported by others in the cell lines HEK 293, U87 and HeLa [71]. All the above changes were eliminated by lithium, trehalose, and NAC + trehalose, and some – by additional drugs used. Given that the above drugs also significantly counteracted all studied rotenone-induced manic-like behavioral paradigms it is tempting to deduce that GRAS autophagy-enhancers and ROS-scavengers are sound candidate anti-manic drugs.

A different pattern of results emerged following eight weeks of rotenone administration which induced a depressive-like behavior. Interestingly, while when rotenone was administered for four weeks it affected (increased) only CoIV, treatment for eight weeks significantly augmented all five mitochondrial complexes’ protein levels and most of the drugs and their combination counteracted rotenone’s effect. As expected, most autophagy-enhancers and ROS-scavengers studied eliminated the increase in most mitochondrial complexes both in the hippocampus and in the frontal-cortex. The drugs also, sporadically, attenuated eight weeks rotenone’s effect on LC3-II and p62 protein levels and their ration, but did not ubiquitously affect rotenone-induced behavioral changes. This raises the possibility that differently from the manic-like behavior, amending depressive–like characteristics requires different mitochondria-related interventions. Given that eliciting depressive–like behavior necessitates twice the duration of exposure to rotenon, and since many antioxidant interventions are preventative in nature, by the time depressive-like signs are apparent, interventions which are preventative are unlikely to be efficacious. At this stage, the kind of molecules that penetrate into the mitochondria or actively transported there might counteract or attenuate rotenone’s effect more easily.

In conclusion, by-and-large, among the autophagy-enhancers and ROS-scavengers tested, lithium is the most effective in counteracting rotenone-induced changes. Trehalose and NAC may also be effective in counteracting the manic-like behavior but, apparently, different mitochondria-related interventions might be necessary to counteract depressive-like behavior, possibly antagonising NMDARs. As for the brain regions, it is expectable that despite the fact that both the frontal-cortex and the hippocampus are involved in the pathophysiology of BD [97–99], the neurochemical results were different among them since they process different informational components [98, 100].

## Supplementary information

Supplementary Material
