# Supplementary material for: Does treatment with autophagy-enhancers and/or ROS-scavengers alleviate behavioral and neurochemical consequences of low-dose rotenone-induced mild mitochondrial dysfunction in mice?
Source: Mol Psychiatry. 2023 Jan 23;28(4):1667–78. doi: 10.1038/s41380-023-01955-x (PMC10208973; doi:10.1038/s41380-023-01955-x)
Supplement: Supplementary file 1 — Supplementary Material [file 41380_2023_1955_MOESM1_ESM.pdf]

Damri *et al* - Supplementary Results

Supplementary table I: **Qualitative effects of the autophagy-enhancers, ROS-scavengers and their combinations on mice' behavior and brain protein levels***

Drugs / Tests	Lithium	Trehalose	NAC	Resveratrol	Lithium+ Resveratrol	NAC+Trehalose
**Behavior**						
FST	↓ 26%	↑ 26%	↔	↓ 24%	↓ 24%	↔
EPM	↑ 32%	↔	↓ 41%	↑ 41%	↔	↓ 29%
SSPT	↔	↔	↓ 42%	↔	↔	↓ 37%
Amphetamine-induced hyperlocomotion	↓ 20%	↓ 21%	↔	↔	↔	↔
**Hippocampal Protein levels**						
Complex I	↔	↑ 119%	↑ 143%	↔	↔	↑ 431%
Complex II	↔	↑ 180%	↔	↔	↔	↑ 300%
Complex III	↔	↑ 102%	↔	↔	↔	↑ 342%
Complex IV	↔	↑ 534%	↔	↔	↔	↑ 476%
Complex V	↔	↑ 259%	↔	↔	↔	↔
LC3-II	↔	↑ 105%	↑ 192%	↔	↔	↔
p62	↔	↔	↔	↔	↔	↔
**Frontal Cortex Protein levels**						
Complex I	↔	↑ 64%	↓ 42%	↓ 49%	↓ 53%	↔
Complex II	↔	↔	↑ 820%	↔	↔	↔
Complex III	↔	↔	↔	↔	↔	↔
Complex IV	↓ 53%	↓ 43%	↔	↔	↔	↓ 48%
Complex V	↓ 41%	↔	↔	↔	↓ 44%	↔
LC3-II	↔	↔	↔	↔	↑ 105%	↓ 192%
p62	↔	↓ 77%	↓ 67%	↔	↔	↔

Percents denote effect *vs*. vehicle
Arrows denote

↔ = No change
↑ = Increase
↓ = Decrease

* Since the controls were administered on their own rather than following rotenone treatment, results obtained from the four and the eight weeks experiments were combined.

The information expands relatively succinct information available concerning the effect of the drugs we selected on specific affective-related behavioral and neurochemical parameters. Evidently, a distinct response to treatment by the various drugs of the hippocampal *vs.* the frontal cortex mitochondrial complexes and autophagy markers protein levels was found.

Supplementary tables of the detailed identification of the Western blot samples and the corresponding original Western blot images*

Treatment with drugs for the last two weeks of the exposure to four weeks of rotenone.

Sample number	Treatment		Sample number	Treatment
100	Vehicle		140	Rotenone
101	Vehicle		141	Rotenone
102	Vehicle		142	Rotenone
103	Vehicle		143	Rotenone
104	Vehicle		144	Rotenone
105	Vehicle		145	Rotenone
106	Vehicle		146	Rotenone
107	Vehicle		147	Rotenone
108	Vehicle		148	Rotenone
109	Vehicle		149	Rotenone
110	Lithium		150	Rotenone+Lithium
111	Lithium		151	Rotenone+Lithium
112	Lithium		152	Rotenone+Lithium
113	Lithium		153	Rotenone+Lithium
114	Lithium		154	Rotenone+Lithium
115	Lithium		155	Rotenone+Lithium
116	Lithium		156	Rotenone+Lithium
117	Lithium		157	Rotenone+Lithium
118	Lithium		158	Rotenone+Lithium
119	Lithium		159	Rotenone+Lithium
120	Resveratrol		160	Rotenone+Resveratrol
121	Resveratrol		161	Rotenone+Resveratrol
122	Resveratrol		162	Rotenone+Resveratrol
123	Resveratrol		163	Rotenone+Resveratrol
124	Resveratrol		164	Rotenone+Resveratrol
125	Resveratrol		165	Rotenone+Resveratrol
126	Resveratrol		166	Rotenone+Resveratrol
127	Resveratrol		167	Rotenone+Resveratrol
128	Resveratrol		168	Rotenone+Resveratrol
129	Resveratrol		169	Rotenone+Resveratrol
130	Lithium+Resveratrol		170	Rotenone+Lithium+Resvertrol
131	Lithium+Resveratrol		171	Rotenone+Lithium+Resvertrol
132	Lithium+Resveratrol		172	Rotenone+Lithium+Resvertrol
133	Lithium+Resveratrol		173	Rotenone+Lithium+Resvertrol
134	Lithium+Resveratrol		174	Rotenone+Lithium+Resvertrol
135	Lithium+Resveratrol		175	Rotenone+Lithium+Resvertrol
136	Lithium+Resveratrol		176	Rotenone+Lithium+Resvertrol
137	Lithium+Resveratrol		178	Rotenone+Lithium+Resvertrol
138	Lithium+Resveratrol		179	Rotenone+Lithium+Resvertrol
139	Lithium+Resveratrol			

Sample Number	Treatment
1	Rotenone+NAC
2	Rotenone+NAC
3	Trehalose
4	Trehalose
5	Trehalose
6	Trehalose
7	Rotenone+Trehalose
8	Rotenone+Trehalose
9	Rotenone+Trehalose
10	Rotenone+Trehalose
11	Rotenone+Trehalose
13	Vehicle
14	Vehicle
15	Vehicle
16	Rotenone
17	Rotenone
18	Rotenone
18	Rotenone
19	Rotenone+NAC
20	Rotenone+NAC
21	NAC
22	NAC
23	NAC
24	NAC
25	NAC
26	NAC
27	Rotenone+Trehalose+NAC
28	Rotenone+Trehalose+NAC
30	Trehalose+NAC
31	Trehalose+NAC
32	Trehalose+NAC
33	Rotenone+Trehalose+NAC
34	Rotenone+Trehalose+NAC
35	Rotenone+Trehalose+NAC
36	Trehalose+NAC
37	Trehalose+NAC
38	Trehalose+NAC

Treatment with drugs for the last two weeks of the exposure to eight weeks of rotenone.

Sample number	Treatment		Sample number	Treatment
1	Vehicle		41	Rotenone+Lithium
2	Vehicle		42	Rotenone+Lithium
3	Vehicle		43	Rotenone+Lithium
4	Vehicle		44	Rotenone+Lithium
5	Vehicle		45	Rotenone+Lithium
6	Vehicle		46	Rotenone+Lithium
7	Vehicle		47	Rotenone+Lithium
8	Vehicle		48	vehicle
9	Lithium		49	vehicle
10	Lithium		50	Li
11	Lithium		51	Li
12	Lithium		52	res
13	Lithium		53	res
14	Lithium		54	Li+res
15	Lithium		55	Li+res
16	Lithium		56	Rotenone
17	Resveratrol		57	Rotenone
18	Resveratrol		58	Rotenone+Lithium
19	Resveratrol		59	Rotenone+Lithium
20	Resveratrol		60	rot+res
21	Resveratrol		61	rot+res
22	Resveratrol		62	Rotenone+Lithium+Resvertrol
23	Resveratrol		63	Rotenone+Lithium+Resvertrol
24	Resveratrol		64	Rotenone+Lithium
25	Lithium+Resveratrol		65	Rotenone+Resveratrol
26	Lithium+Resveratrol		66	Rotenone+Resveratrol
27	Lithium+Resveratrol		67	Rotenone+Resveratrol
28	Lithium+Resveratrol		68	Rotenone+Resveratrol
29	Lithium+Resveratrol		69	Rotenone+Resveratrol
30	Lithium+Resveratrol		70	Rotenone+Resveratrol
31	Lithium+Resveratrol		71	Rotenone+Resveratrol
32	Lithium+Resveratrol		72	Rotenone+Resveratrol
33	Rotenone		73	Rotenone+Lithium+Resvertrol
34	Rotenone		74	Rotenone+Lithium+Resvertrol
35	Rotenone		75	Rotenone+Lithium+Resvertrol
36	Rotenone		76	Rotenone+Lithium+Resvertrol
37	Rotenone		77	Rotenone+Lithium+Resvertrol
38	Rotenone		78	Rotenone+Lithium+Resvertrol
39	Rotenone		79	Rotenone+Lithium+Resvertrol
40	Rotenone		80	Rotenone+Lithium+Resvertrol

Sample Number	Treatment
1	Vehicle
2	Vehicle
3	Vehicle
4	Vehicle
5	Rotenone
6	Rotenone
7	Rotenone
8	Rotenone
9	Rotenone
10	NAC
11	NAC
12	NAC
13	NAC
14	NAC
15	Rotenone+NAC
16	Rotenone+NAC
17	Rotenone+NAC
18	Rotenone+NAC
19	Rotenone+NAC
20	Trehalose
21	Trehalose
22	Trehalose
23	Trehalose
24	Trehalose
25	Rotenone+Trehalose
26	Rotenone+Trehalose
27	Rotenone+Trehalose
28	Rotenone+Trehalose
29	Trehalose+NAC
30	Trehalose+NAC
31	Trehalose+NAC
32	Trehalose+NAC
33	Trehalose+NAC
34	Rotenone+Trehalose+NAC
35	Rotenone+Trehalose+NAC
36	Rotenone+Trehalose+NAC
37	Rotenone+Trehalose+NAC
38	Rotenone+Trehalose+NAC

Gels design Hippocampus

gel -1 ox HP														
leader	100	100	110	110	120	120	130	130	140	140	150	150	160	160
gel -2 ox HP														
170	170	101	101	111	111	121	121	leader	171	171	161	161	141	141
gel-3 ox HP														
leader	102	102	112	112	122	122	131	131	142	142	151	151	162	162
gel-4 oxHP														
172	172	169	169	152	152	143	143	leader	132	132	123	123	113	113
gel-5 ox HP														
leader	103	103	114	114	124	124	133	133	144	144	153	153	163	163
gel-6 ox HP														
173	173	164	164	154	154	145	145	leader	134	134	125	125	115	115
gel-7 ox HP														
leader	104	104	116	116	126	126	135	135	146	146	155	155	165	165
gel-8 ox HP														
174	174	166	166	156	156	147	147	leader	136	136	127	127	117	117
gel-9 ox HP														
leader	105	105	106	106	118	118	137	137	168	168	175	175	176	176
gel-10 ox HP														
178	178	179	179	167	167	157	157	leader	148	148	138	138	128	128
gel-11 ox HP														
leader	107	107	108	108	119	119	159	159	139	139	149	149	158	158
gel-12 ox HP														
109	109	129	129	leader	177	177								

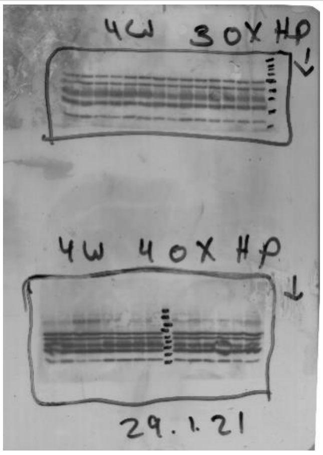

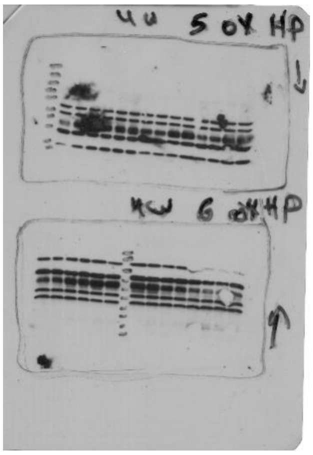

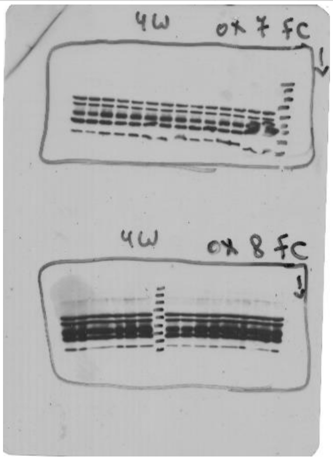

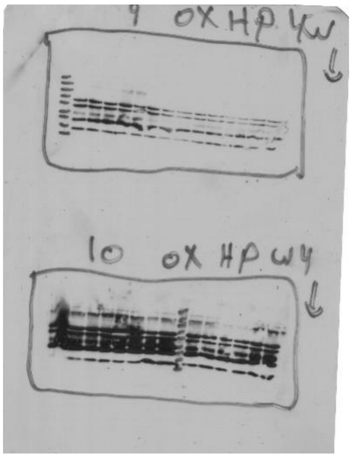

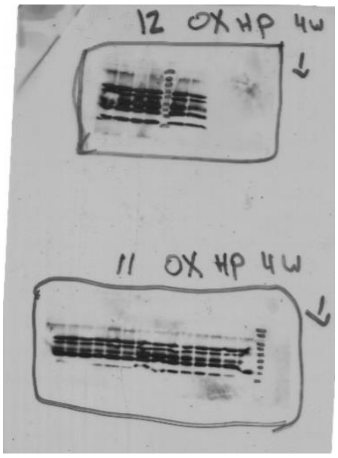

Gels design Frontal Cortex

							gel -1 ox							
leader	100	100	110	110	120	120	130	130	140	140	150	150	160	160
							gel -2 ox							
170	170	101	101	111	111	121	121	leader	171	171	161	161	141	141
							gel-3 ox							
leader	102	102	112	112	122	122	131	131	142	142	151	151	162	162
							gel-4 ox							
172	172	169	169	152	152	143	143	leader	132	132	123	123	113	113
							gel-5 ox							
leader	103	103	114	114	124	124	133	133	144	144	153	153	163	163
							gel-6 ox							
173	173	164	164	154	154	145	145	leader	134	134	125	125	115	115
							gel-7 ox							
leader	104	104	116	116	126	126	135	135	146	146	155	155	165	165
							gel-8 ox							
174	174	166	166	156	156	147	147	leader	136	136	127	127	117	117
							gel-9 ox							
leader	105	105	106	106	118	118	137	137	168	168	175	175	176	176
							gel-10 ox							
178	178	179	179	167	167	157	157	leader	148	148	138	138	128	128
							gel-11 ox							
leader	107	107	108	108	119	119	159	159	139	139	149	149	158	158
							gel-12 ox							
109	109	129	129	leader	177	177								

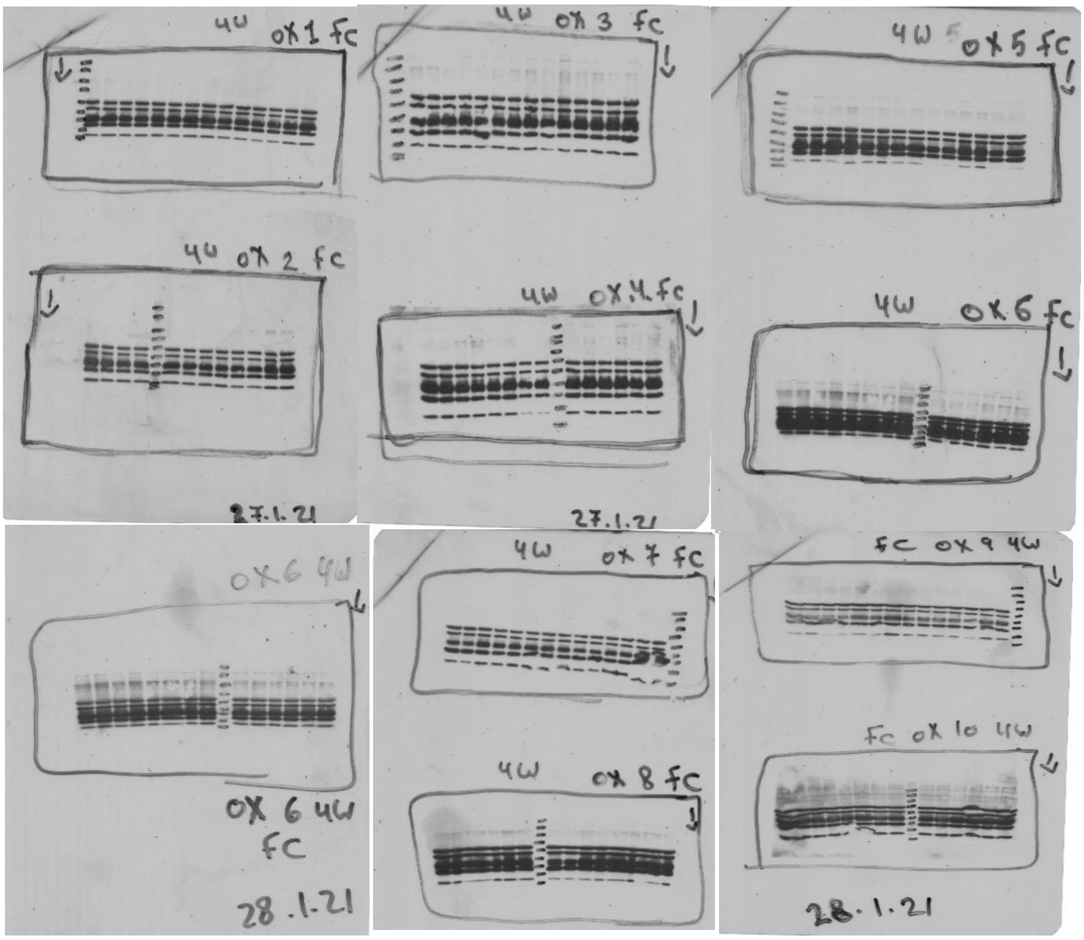

Gel design Hippocampus

gel 21 OX													
10µl							20µl						
13	16	14	17	15	18	21	13	16	14	17	15	18	21
gel 24 OX													
10µl							20µl						
13	22	1	23	2	24	19	13	22	1	23	2	24	19
gel 27 OX													
10µl							20µl						
13	25	26	20	3	7	4	13	25	26	20	3	7	4
gel 30 OX													
10µl							20µl						
13	8	5	9	6	10	11	13	8	5	9	6	10	11
gel 33 OX													
10µl							20µl						
13	30	27	31	28	32	29	13	30	27	31	28	32	29
gel 36 OX													
10µl							20µl						
13	33	36	37	34	38	35	13	33	36	37	34	38	35

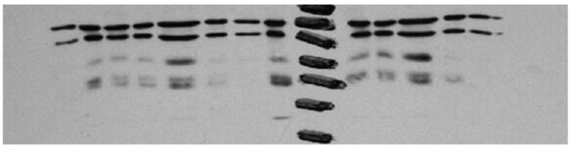

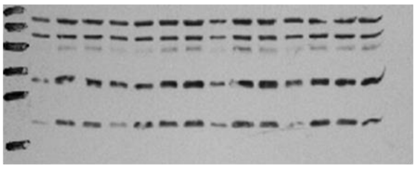

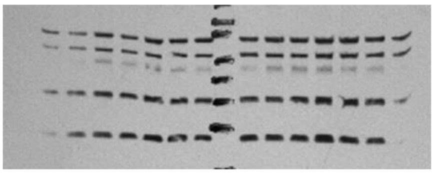

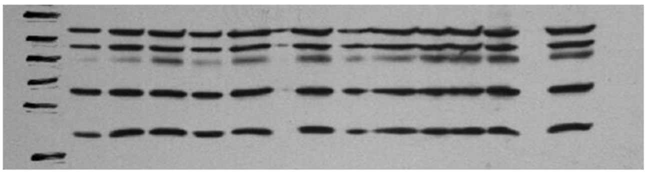

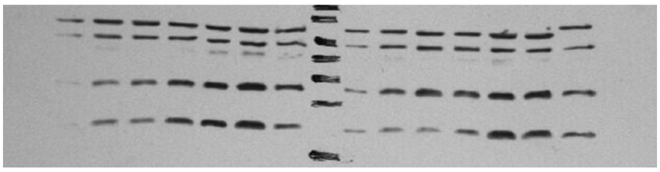

Gel design Frontal Cortex

gel 3 OX													
10µl							20µl						
13	16	14	17	15	18	21	13	16	14	17	15	18	21
gel 6 OX													
10µl							20µl						
13	22	1	23	2	24	19	13	22	1	23	2	24	19
gel 9 OX													
10µl							20µl						
13	25	26	20	3	7	4	13	25	26	20	3	7	4
gel 12 OX													
10µl							20µl						
13	8	5	9	6	10	11	13	8	5	9	6	10	11

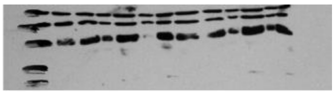

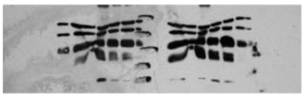

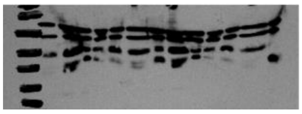

Gels design Hippocampus

gel -1 HP ox 8W														
leader	1	1	9	9	17	17	23	23	27	27	32	32	40	40
gel -2 HP ox 8W														
80	80	71	71	66	66	43	43	leader	28	28	21	21	11	11
gel-3														
leader	2	2	10	10	18	18	29	29	38	38	47	47	76	76
gel-4														
3	3	79	79	72	72	42	42	leader	33	33	19	19	7	7
gel-5														
leader	5	5	35	35	30	30	20	20	8	8	77	77	64	64
gel-6														
45	45	26	26	6	6	13	13	leader	37	37	41	41	39	39
gel-7														
leader	22	22	68	68	69	69	46	46	12	12	65	65	15	15
gel-8														
31	31	16	16	25	25	36	36	leader	44	44	37	37	14	14

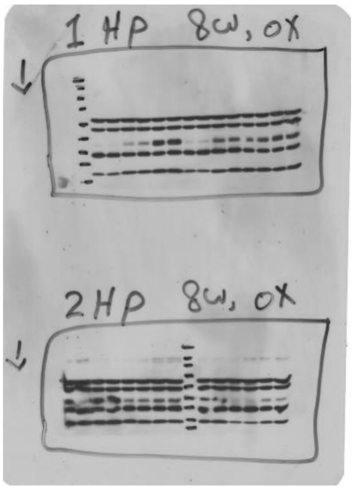

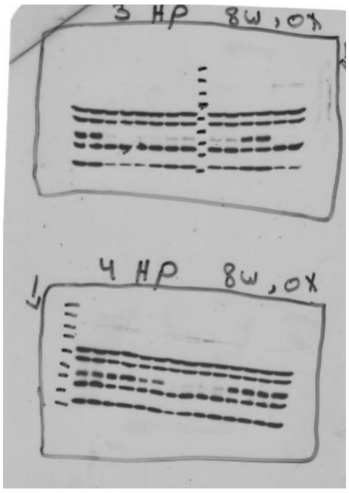

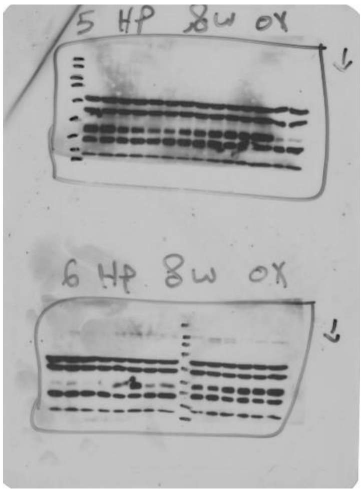

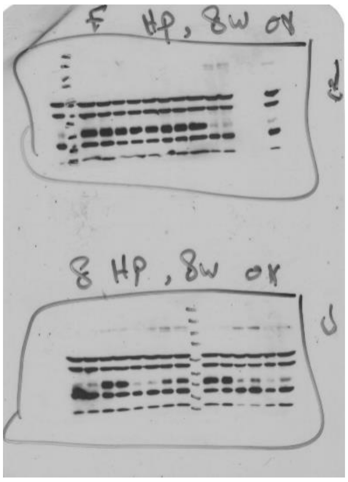

Gels design Frontal Cortex

gel -1 ox 8W														
leader	1	1	9	9	17	17	23	23	27	27	32	32	40	40
gel -2 ox														
leader	80	80	71	71	66	66	43	43	28	28	21	21	11	11
gel-3 ox														
2	2	10	10	18	18	29	29	leader	38	38	47	47	76	76
gel-4 ox														
3	3	79	79	72	72	42	42	leader	33	33	19	19	7	7
gel-5 ox														
leader	5	5	35	35	30	30	20	20	8	8	77	77	64	64
gel-6 ox														
leader	45	45	26	26	6	6	13	13	37	37	41	41	39	39
gel-7 ox														
22	22	68	68	69	69	46	46	leader	12	12	65	65	15	15
gel-8 ox														
Leader	31	31	16	16	25	25	36	36	44	44	37	37	14	14
gel-9 ox														
67	67	24	24	74	74	4	4	leader	78	78	70	70	34	34

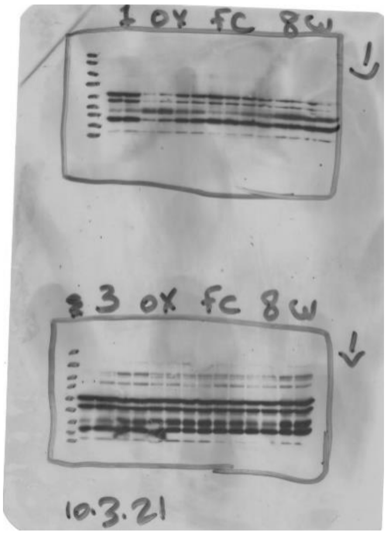

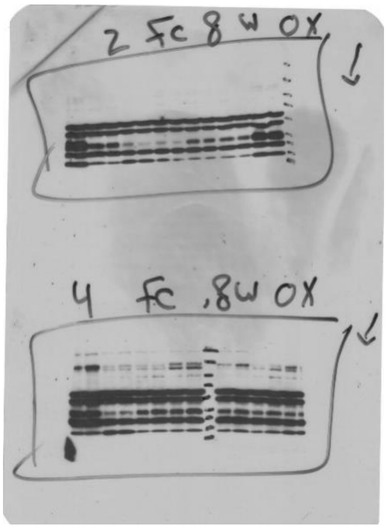

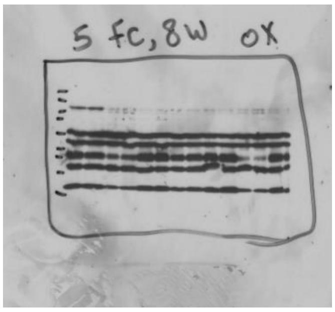

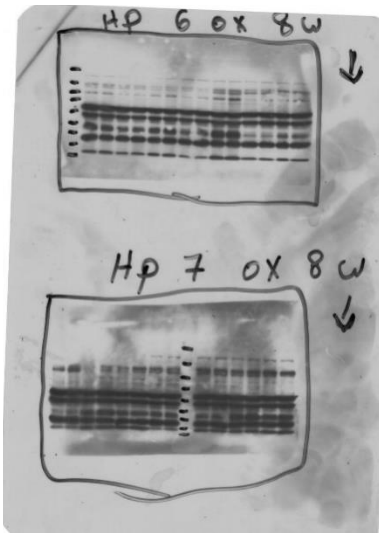

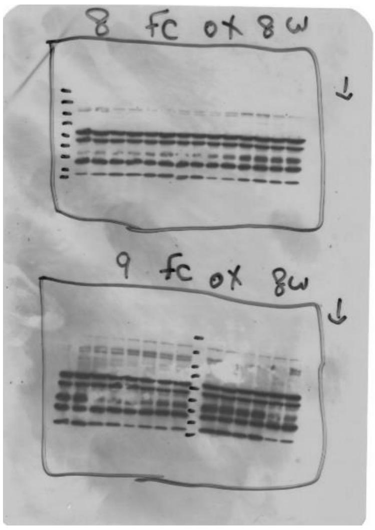

Gels design Hippocampus

gel 21 OX													
10µl							20µl						
1	5	2	6	3	7	9	1	5	2	6	3	7	9
gel 24 OX													
10µl							20µl						
1	4	8	10	15	11	16	1	4	8	10	15	11	16
gel 27 OX													
10µl							20µl						
1	12	17	13	18	14	19	1	12	17	13	18	14	19
gel 30 OX													
10µl							20µl						
1	20	25	21	26	22	27	1	20	25	21	26	22	27
gel 33 OX													
10µl							20µl						
1	23	28	24	29	34	30	1	23	28	24	29	34	30
gel 36 OX													
10µl							20µl						
1	35	31	36	32	37	33	1	35	31	36	32	37	33

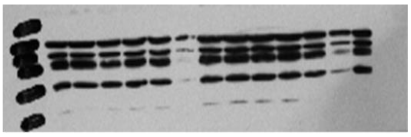

Gel 21

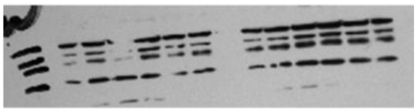

Gel 24

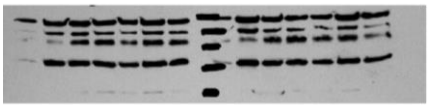

Gel 27

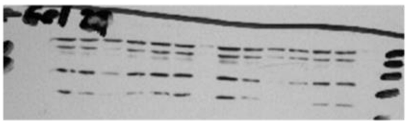

Gel 30

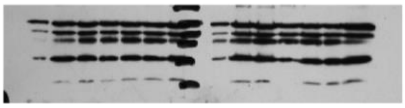

Gel 33

Gels design Frontal cortex

gel 3 OX													
10µl							20µl						
1	5	2	6	3	7	9	1	5	2	6	3	7	9
gel 6 OX													
10µl							20µl						
1	4	8	10	15	11	16	1	4	8	10	15	11	16
gel 9 OX													
10µl							20µl						
1	12	17	13	18	14	19	1	12	17	13	18	14	19
gel 12 OX													
10µl							20µl						
1	20	25	21	26	22	27	1	20	25	21	26	22	27
gel 15 OX													
10µl							20µl						
1	23	28	24	29	34	30	1	23	28	24	29	34	30
gel 18 OX													
10µl							20µl						
1	35	31	36	32	37	33	1	35	31	36	32	37	33

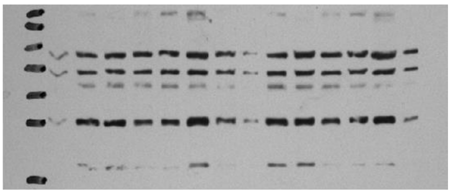

Gel 3

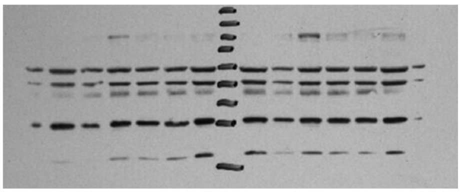

Gel 6

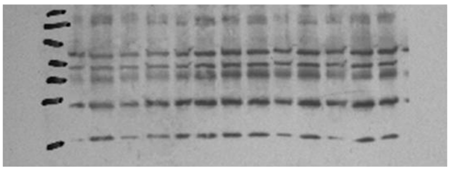

Gel 9

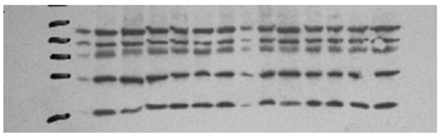

Gel 12

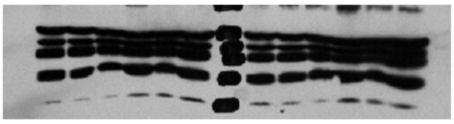

Gel 15

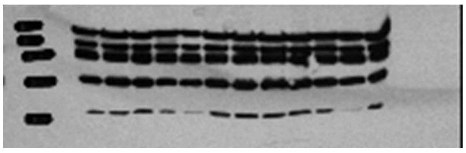

Gel 18

<u>Treatment with drugs for the last two weeks of the exposure to four weeks of rotenone.</u>

Sample number	Treatment		Sample number	Treatment
100	Vehicle		140	Rotenone
101	Vehicle		141	Rotenone
102	Vehicle		142	Rotenone
103	Vehicle		143	Rotenone
104	Vehicle		144	Rotenone
105	Vehicle		145	Rotenone
106	Vehicle		146	Rotenone
107	Vehicle		147	Rotenone
108	Vehicle		148	Rotenone
109	Vehicle		149	Rotenone
110	Lithium		150	Rotenone+Lithium
111	Lithium		151	Rotenone+Lithium
112	Lithium		152	Rotenone+Lithium
113	Lithium		153	Rotenone+Lithium
114	Lithium		154	Rotenone+Lithium
115	Lithium		155	Rotenone+Lithium
116	Lithium		156	Rotenone+Lithium
117	Lithium		157	Rotenone+Lithium
118	Lithium		158	Rotenone+Lithium
119	Lithium		159	Rotenone+Lithium
120	Resveratrol		160	Rotenone+Resveratrol
121	Resveratrol		161	Rotenone+Resveratrol
122	Resveratrol		162	Rotenone+Resveratrol
123	Resveratrol		163	Rotenone+Resveratrol
124	Resveratrol		164	Rotenone+Resveratrol
125	Resveratrol		165	Rotenone+Resveratrol
126	Resveratrol		166	Rotenone+Resveratrol
127	Resveratrol		167	Rotenone+Resveratrol
128	Resveratrol		168	Rotenone+Resveratrol
129	Resveratrol		169	Rotenone+Resveratrol
130	Lithium+Resveratrol		170	Rotenone+Lithium+Resvertrol
131	Lithium+Resveratrol		171	Rotenone+Lithium+Resvertrol
132	Lithium+Resveratrol		172	Rotenone+Lithium+Resvertrol
133	Lithium+Resveratrol		173	Rotenone+Lithium+Resvertrol
134	Lithium+Resveratrol		174	Rotenone+Lithium+Resvertrol
135	Lithium+Resveratrol		175	Rotenone+Lithium+Resvertrol
136	Lithium+Resveratrol		176	Rotenone+Lithium+Resvertrol
137	Lithium+Resveratrol		178	Rotenone+Lithium+Resvertrol
138	Lithium+Resveratrol		179	Rotenone+Lithium+Resvertrol
139	Lithium+Resveratrol			

Sample Number	Treatment
1	Rotenone+NAC
2	Rotenone+NAC
3	Trehalose
4	Trehalose
5	Trehalose
6	Trehalose
7	Rotenone+Trehalose
8	Rotenone+Trehalose
9	Rotenone+Trehalose
10	Rotenone+Trehalose
11	Rotenone+Trehalose
13	Vehicle
14	Vehicle
15	Vehicle
16	Rotenone
17	Rotenone
18	Rotenone
18	Rotenone
19	Rotenone+NAC
20	Rotenone+NAC
21	NAC
22	NAC
23	NAC
24	NAC
25	NAC
26	NAC
27	Rotenone+Trehalose+NAC
28	Rotenone+Trehalose+NAC
30	Trehalose+NAC
31	Trehalose+NAC
32	Trehalose+NAC
33	Rotenone+Trehalose+NAC
34	Rotenone+Trehalose+NAC
35	Rotenone+Trehalose+NAC
36	Trehalose+NAC
37	Trehalose+NAC
38	Trehalose+NAC

Treatment with drugs for the last two weeks of the exposure to eight weeks of rotenone.

Sample number	Treatment		Sample number	Treatment
1	Vehicle		41	Rotenone+Lithium
2	Vehicle		42	Rotenone+Lithium
3	Vehicle		43	Rotenone+Lithium
4	Vehicle		44	Rotenone+Lithium
5	Vehicle		45	Rotenone+Lithium
6	Vehicle		46	Rotenone+Lithium
7	Vehicle		47	Rotenone+Lithium
8	Vehicle		48	vehicle
9	Lithium		49	vehicle
10	Lithium		50	Li
11	Lithium		51	Li
12	Lithium		52	res
13	Lithium		53	res
14	Lithium		54	Li+res
15	Lithium		55	Li+res
16	Lithium		56	Rotenone
17	Resveratrol		57	Rotenone
18	Resveratrol		58	Rotenone+Lithium
19	Resveratrol		59	Rotenone+Lithium
20	Resveratrol		60	rot+res
21	Resveratrol		61	rot+res
22	Resveratrol		62	Rotenone+Lithium+Resvertrol
23	Resveratrol		63	Rotenone+Lithium+Resvertrol
24	Resveratrol		64	Rotenone+Lithium
25	Lithium+Resveratrol		65	Rotenone+Resveratrol
26	Lithium+Resveratrol		66	Rotenone+Resveratrol
27	Lithium+Resveratrol		67	Rotenone+Resveratrol
28	Lithium+Resveratrol		68	Rotenone+Resveratrol
29	Lithium+Resveratrol		69	Rotenone+Resveratrol
30	Lithium+Resveratrol		70	Rotenone+Resveratrol
31	Lithium+Resveratrol		71	Rotenone+Resveratrol
32	Lithium+Resveratrol		72	Rotenone+Resveratrol
33	Rotenone		73	Rotenone+Lithium+Resvertrol
34	Rotenone		74	Rotenone+Lithium+Resvertrol
35	Rotenone		75	Rotenone+Lithium+Resvertrol
36	Rotenone		76	Rotenone+Lithium+Resvertrol
37	Rotenone		77	Rotenone+Lithium+Resvertrol
38	Rotenone		78	Rotenone+Lithium+Resvertrol
39	Rotenone		79	Rotenone+Lithium+Resvertrol
40	Rotenone		80	Rotenone+Lithium+Resvertrol

Sample Number	Treatment
1	Vehicle
2	Vehicle
3	Vehicle
4	Vehicle
5	Rotenone
6	Rotenone
7	Rotenone
8	Rotenone
9	Rotenone
10	NAC
11	NAC
12	NAC
13	NAC
14	NAC
15	Rotenone+NAC
16	Rotenone+NAC
17	Rotenone+NAC
18	Rotenone+NAC
19	Rotenone+NAC
20	Trehalose
21	Trehalose
22	Trehalose
23	Trehalose
24	Trehalose
25	Rotenone+Trehalose
26	Rotenone+Trehalose
27	Rotenone+Trehalose
28	Rotenone+Trehalose
29	Trehalose+NAC
30	Trehalose+NAC
31	Trehalose+NAC
32	Trehalose+NAC
33	Trehalose+NAC
34	Rotenone+Trehalose+NAC
35	Rotenone+Trehalose+NAC
36	Rotenone+Trehalose+NAC
37	Rotenone+Trehalose+NAC
38	Rotenone+Trehalose+NAC

Gels design Hippocampus for LC3II and p62

gel -1 lc3+p62														
leader	100	100	110	110	120	120	130	130	140	140	150	150	160	160
gel -2 lc3+p62														
170	170	101	101	111	111	121	121	leader	171	171	161	161	141	141
gel-3 lc3+p62														
leader	102	102	112	112	122	122	131	131	142	142	151	151	162	162
gel-4 lc3+p62														
172	172	169	169	152	152	143	143	leader	132	132	123	123	113	113
gel-5 lc3+p62														
leader	103	103	114	114	124	124	133	133	144	144	153	153	163	163
gel-6 lc3+p62														
173	173	164	164	154	154	145	145	leader	134	134	125	125	115	115
gel-7 lc3+p62														
leader	104	104	116	116	126	126	135	135	146	146	155	155	165	165
gel-8 lc3+p62														
174	174	166	166	156	156	147	147	leader	136	136	127	127	117	117
gel-9 lc3+p62														
leader	105	105	106	106	118	118	137	137	168	168	175	175	176	176
gel-10 lc3+p62														
178	178	179	179	167	167	157	157	leader	148	148	138	138	128	128
gel-11 lc3+p62														
leader	107	107	108	108	119	119	159	159	139	139	149	149	158	158
gel-12 lc3+p62														
109	109	129	129	leader	177	177								

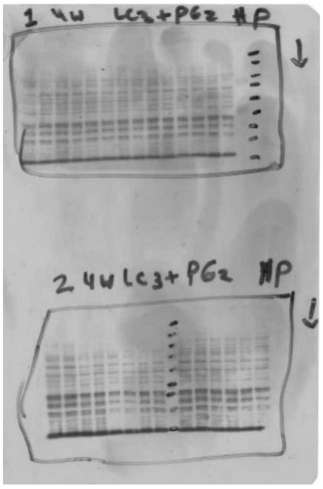

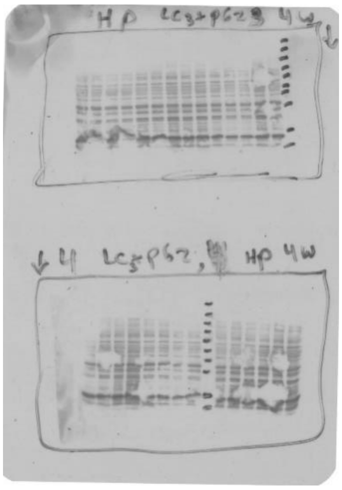

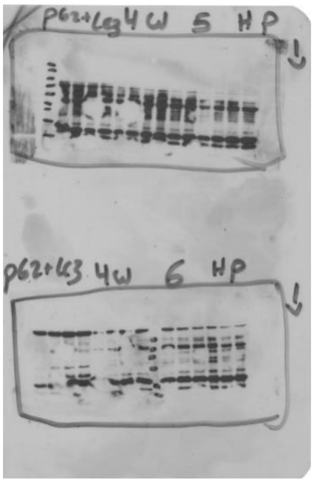

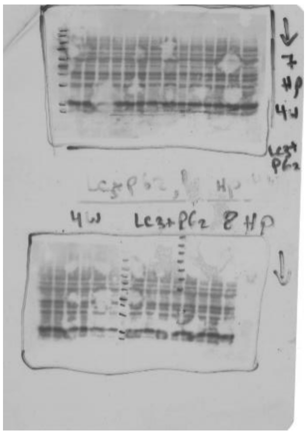

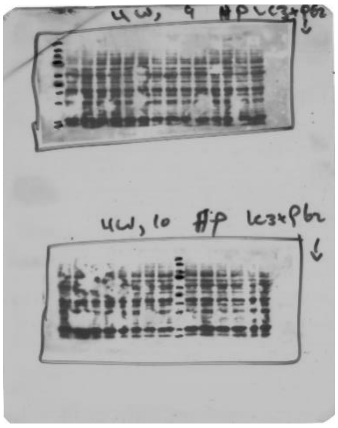

Gels design Frontal Cortex for LC3II and p62

gel -1 lc3+p62														
leader	100	100	110	110	120	120	130	130	140	140	150	150	160	160
gel -2 lc3+p62														
170	170	101	101	111	111	121	121	leader	171	171	161	161	141	141
gel-3 lc3+p62														
leader	102	102	112	112	122	122	131	131	142	142	151	151	162	162
gel-4 lc3+p62														
172	172	169	169	152	152	143	143	leader	132	132	123	123	113	113
gel-5 lc3+p62														
leader	103	103	114	114	124	124	133	133	144	144	153	153	163	163
gel-6 lc3+p62														
173	173	164	164	154	154	145	145	leader	134	134	125	125	115	115
gel-7 lc3+p62														
leader	104	104	116	116	126	126	135	135	146	146	155	155	165	165
gel-8 lc3+p62														
174	174	166	166	156	156	147	147	leader	136	136	127	127	117	117
gel-9 lc3+p62														
leader	105	105	106	106	118	118	137	137	168	168	175	175	176	176
gel-10 lc3+p62														
178	178	179	179	167	167	157	157	leader	148	148	138	138	128	128
gel-11 lc3+p62														
leader	107	107	108	108	119	119	159	159	139	139	149	149	158	158
gel-12 lc3+p62														
109	109	129	129	leader	177	177								

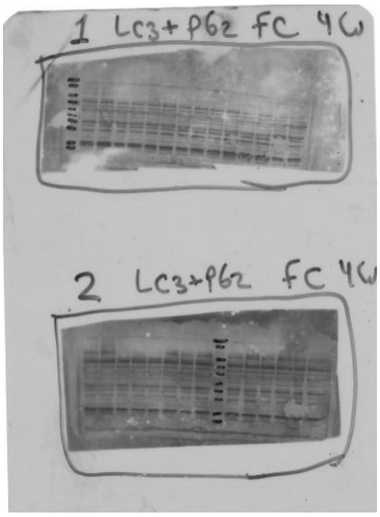

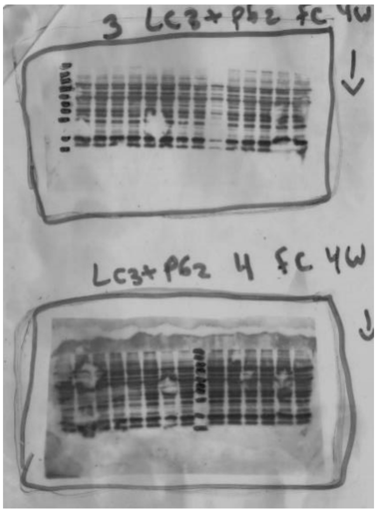

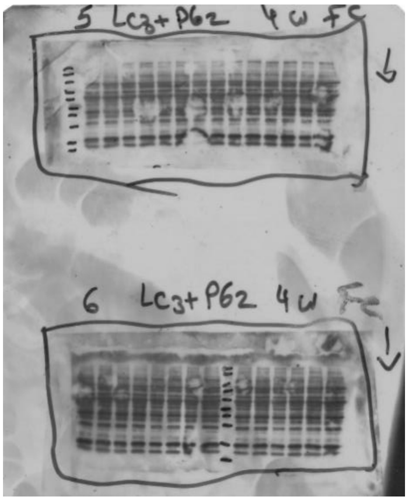

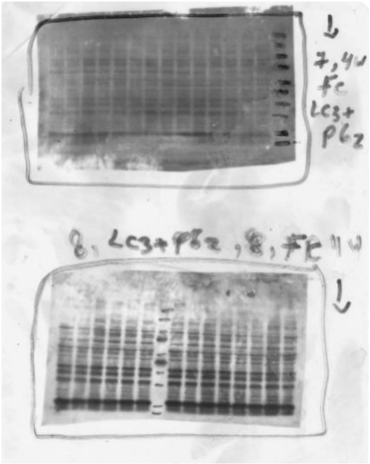

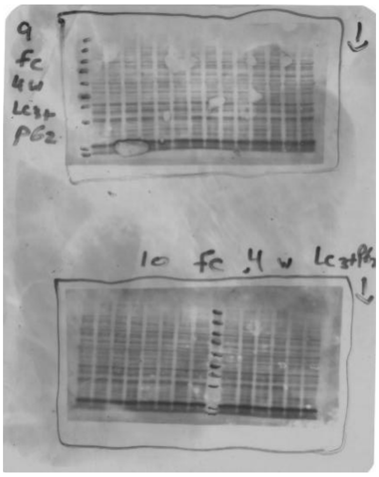

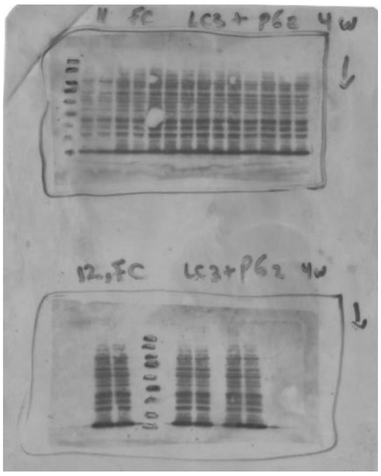

Gels design Hippocampus for LC3II

gel 19 LC3													
10µl							20µl						
13	16	14	17	15	18	21	13	16	14	17	15	18	21
gel 22 LC32													
10µl							20µl						
13	22	1	23	2	24	19	13	22	1	23	2	24	19
gel 25LC32													
10µl							20µl						
13	25	26	20	3	7	4	13	25	26	20	3	7	4
gel 28 LC32													
10µl							20µl						
13	8	5	9	6	10	11	13	8	5	9	6	10	11
gel 31 LC32													
10µl							20µl						
13	30	27	31	28	32	29	13	30	27	31	28	32	29
gel 34 LC32													
10µl							20µl						
13	33	36	37	34	38	35	13	33	36	37	34	38	35

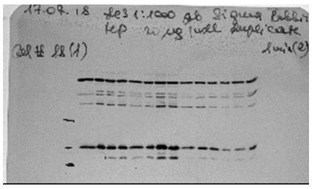
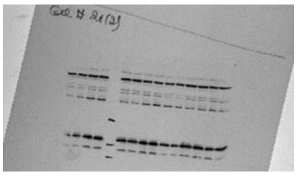
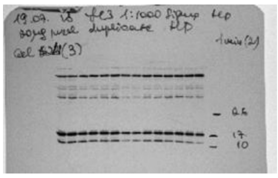
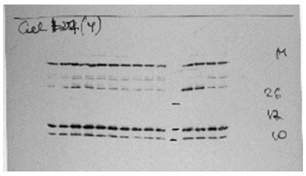
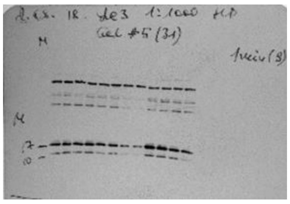
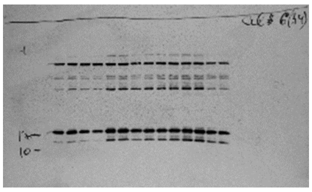

Gels design Hippocampus for p62

gel 20 P62													
10µl							20µl						
13	16	14	17	15	18	21	13	16	14	17	15	18	21
gel 23 P62													
10µl							20µl						
13	22	1	23	2	24	19	13	22	1	23	2	24	19
gel 26 P62													
10µl							20µl						
13	25	26	20	3	7	4	13	25	26	20	3	7	4
gel 29 P62													
10µl							20µl						
13	8	5	9	6	10	11	13	8	5	9	6	10	11
gel 32 P62													
10µl							20µl						
13	30	27	31	28	32	29	13	30	27	31	28	32	29
gel 35 P62													
10µl							20µl						
13	33	36	37	34	38	35	13	33	36	37	34	38	35

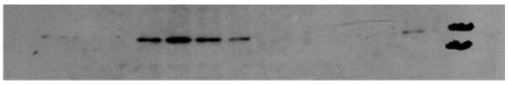

Gel 20

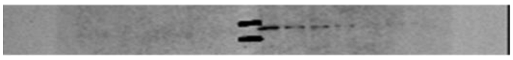

Gel 23

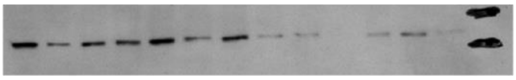

Gel 26

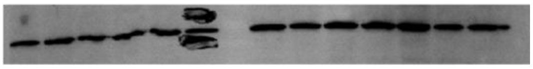

Gel 29

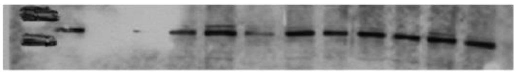

Gel 32

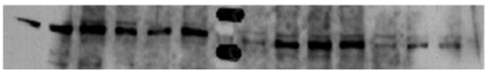

Gel 35

Gels design Frontal Cortex for LC3II

gel 1 LC32													
10µl							20µl						
13	16	14	17	15	18	21	13	16	14	17	15	18	21
gel 4 LC32													
10µl							20µl						
13	22	1	23	2	24	19	13	22	1	23	2	24	19
gel 7 LC32													
10µl							20µl						
13	25	26	20	3	7	4	13	25	26	20	3	7	4
gel 10 LC32													
10µl							20µl						
13	8	5	9	6	10	11	13	8	5	9	6	10	11
gel 13 LC32													
10µl							20µl						
13	30	27	31	28	32	29	13	30	27	31	28	32	29
gel 16 LC32													
10µl							20µl						
13	33	36	37	34	38	35	13	33	36	37	34	38	35

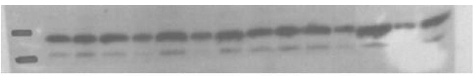

Gel 1

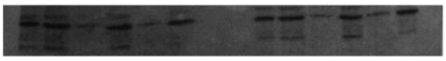

Gel 4

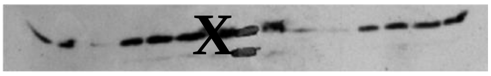

Gel 7

Discarded

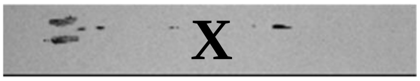

Gel 10

Discarded

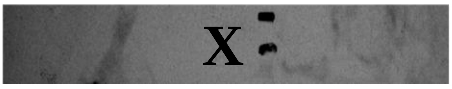

Gel 13

Discarded

Gels design Frontal Cortex for p62

gel 2 P62													
10µl							20µl						
13	16	14	17	15	18	21	13	16	14	17	15	18	21
gel 5 P62													
10µl							20µl						
13	22	1	23	2	24	19	13	22	1	23	2	24	19
gel 8 P62													
10µl							20µl						
13	25	26	20	3	7	4	13	25	26	20	3	7	4
gel 11 P62													
10µl							20µl						
13	8	5	9	6	10	11	13	8	5	9	6	10	11
gel 14 P62													
10µl							20µl						
13	30	27	31	28	32	29	13	30	27	31	28	32	29
gel 17 p62													
10µl							20µl						
13	33	36	37	34	38	35	13	33	36	37	34	38	35

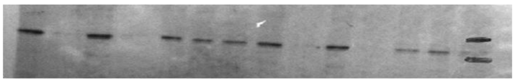

Gel 2

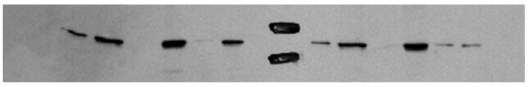

Gel 5

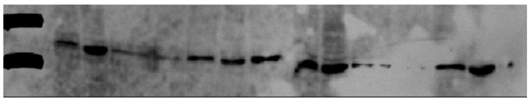

Gel 8

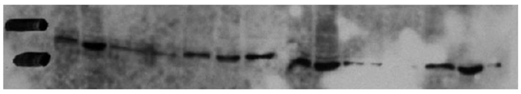

Gel 11

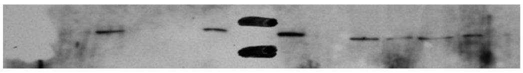

Gel 14

Gels design Hippocampus for LC3II

gel 19 LC32													
10µl							20µl						
1	5	2	6	3	7	9	1	5	2	6	3	7	9
gel 22 LC32													
10µl							20µl						
1	4	8	10	15	11	16	1	4	8	10	15	11	16
gel 25 LC32													
10µl							20µl						
1	12	17	13	18	14	19	1	12	17	13	18	14	19
gel 28 LC32													
10µl							20µl						
1	20	25	21	26	22	27	1	20	25	21	26	22	27
gel 31 LC32													
10µl							20µl						
1	23	28	24	29	34	30	1	23	28	24	29	34	30
gel 34 LC32													
10µl							20µl						
1	35	31	36	32	37	33	1	35	31	36	32	37	33
gel 25 LC32													
10µl							20µl						
1	12	17	13	18	14	19	1	12	17	13	18	14	19
gel 31 LC32													
10µl							20µl						
1	23	28	24	29	34	30	1	23	28	24	29	34	30
gel 34 LC32													
10µl							20µl						
1	35	31	36	32	37	33	1	35	31	36	32	37	33

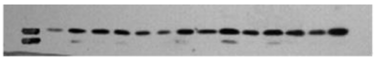

Gel 19

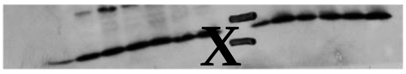

Gel 34

Discarded

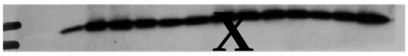

Gel 31

Discarded

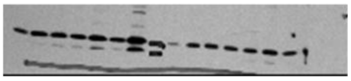

Gel 22

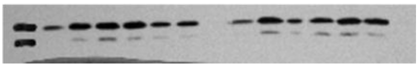

Gel 25

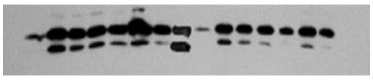

Gel 28

Gels design Hippocampus for p62

gel20 P62													
10µl							20µl						
1	5	2	6	3	7	9	1	5	2	6	3	7	9
gel 23 P62													
10µl							20µl						
1	4	8	10	15	11	16	1	4	8	10	15	11	16
gel 26 P62													
10µl							20µl						
1	12	17	13	18	14	19	1	12	17	13	18	14	19
gel 29 P62													
10µl							20µl						
1	20	25	21	26	22	27	1	20	25	21	26	22	27
gel 32 P62													
10µl							20µl						
1	23	28	24	29	34	30	1	23	28	24	29	34	30
gel 35 P62													
10µl							20µl						
1	35	31	36	32	37	33	1	35	31	36	32	37	33
gel 29 P62													
10µl							20µl						
1	20	25	21	26	22	27	1	20	25	21	26	22	27

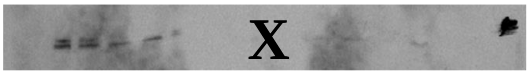

Gel 14

Discarded

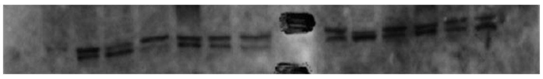

Gel 17

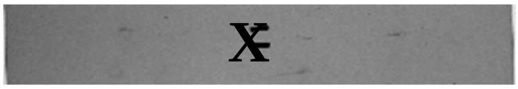

Gel 29

Discarded

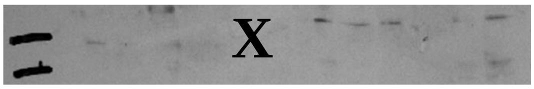

Gel 32

Discarded

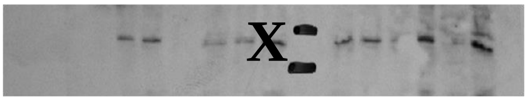

Gel 35

Discarded

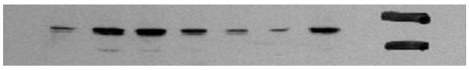

Gel 20

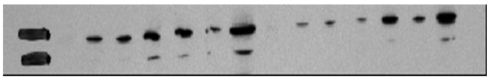

Gel 23

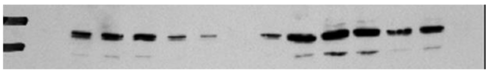

Gel 26

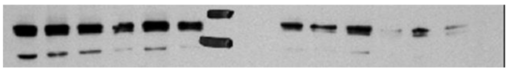

Gel 29

Gels design Frontal Cortex for LC3II

gel 1 lc3													
10µl							20µl						
1	5	2	6	3	7	9	1	5	2	6	3	7	9
gel 4 LC32													
10µl							20µl						
1	4	8	10	15	11	16	1	4	8	10	15	11	16
gel 7 LC32													
10µl							20µl						
1	12	17	13	18	14	19	1	12	17	13	18	14	19
gel 10 LC32													
10µl							20µl						
1	20	25	21	26	22	27	1	20	25	21	26	22	27
gel 13 LC32													
10µl							20µl						
1	23	28	24	29	34	30	1	23	28	24	29	34	30
gel 16 LC32													
10µl							20µl						
1	35	31	36	32	37	33	1	35	31	36	32	37	33

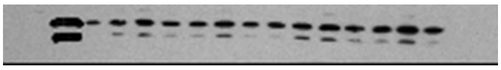

Gel 1

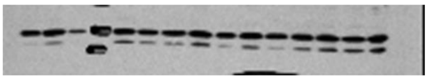

Gel 4

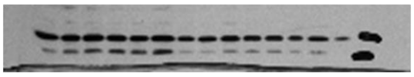

Gel 7

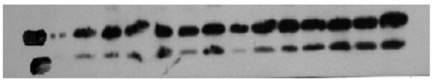

Gel 10

Gels design Frontal Cortex for p62

gel 2 P62													
10µl							20µl						
1	5	2	6	3	7	9	1	5	2	6	3	7	9
gel 5 P62													
10µl							20µl						
1	4	8	10	15	11	16	1	4	8	10	15	11	16
gel 8 P62													
10µl							20µl						
1	12	17	13	18	14	19	1	12	17	13	18	14	19
gel 11 P62													
10µl							20µl						
1	20	25	21	26	22	27	1	20	25	21	26	22	27
gel 14 P62													
10µl							20µl						
1	23	28	24	29	34	30	1	23	28	24	29	34	30
gel 17 P62													
10µl							20µl						
1	35	31	36	32	37	33	1	35	31	36	32	37	33

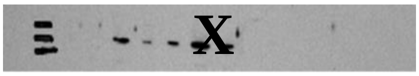

Gel 2

Discarded

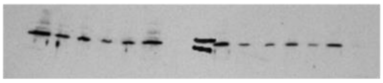

Gel 5

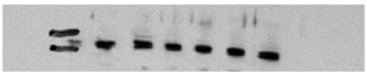

Gel 8

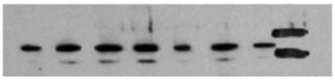

Gel 11

*The original Western blot images from which Figs. 3-5 were extracted.
